# Embryonic Program Activated during Blast Crisis of Chronic Myelogenous Leukemia (CML) Implicates a TCF7L2 and MYC Cooperative Chromatin Binding

**DOI:** 10.3390/ijms21114057

**Published:** 2020-06-05

**Authors:** Christophe Desterke, Patricia Hugues, Jin Wook Hwang, Annelise Bennaceur-Griscelli, Ali G. Turhan

**Affiliations:** 1 INSERM U935, University Paris Saclay, 94802 Villejuif, France; christophe.desterke@gmail.com (C.D.); patricia.hugues@u-psud.fr (P.H.); jinwook.hwang@inserm.fr (J.W.H.); abenna@hotmail.fr (A.B.-G.); 2 APHP Paris Saclay, University Paris Saclay, 94802 Villejuif, France; 3 INGESTEM Pluripotent Stem Cell Core Facility, 94802 Villejuif, France

**Keywords:** chronic myelogenous leukemia, epigenetics, blast crisis, MYC, TCF7L2, leukemic stem cells

## Abstract

Chronic myeloid leukemia (CML) is characterized by an inherent genetic instability, which contributes to the progression of the disease towards an accelerated phase (AP) and blast crisis (BC). Several cytogenetic and genomic alterations have been reported in the progression towards BC, but the precise molecular mechanisms of this event are undetermined. Transcription Factor 7 like 2 (TFC7L2) is a member of the TCF family of proteins that are known to activate WNT target genes such as Cyclin D1. TCF7L2 has been shown to be overexpressed in acute myeloid leukemia (AML) and represents a druggable target. We report here that TCF7L2 transcription factor expression was found to be correlated to blast cell numbers during the progression of the disease. In these cells, TCF7L2 CHIP-sequencing highlighted distal cis active enhancer, such as elements in SMAD3, ATF5, and PRMT1 genomic regions and a proximal active transcriptional program of 144 genes. The analysis of CHIP-sequencing of MYC revealed a significant overlapping of TCF7L2 epigenetic program with MYC. The β-catenin activator lithium chloride and the MYC-MAX dimerization inhibitor 10058-F4 significantly modified the expression of three epigenetic targets in the BC cell line K562. These results suggest for the first time the cooperative role of TCF7L2 and MYC during CML-BC and they strengthen previous data showing a possible involvement of embryonic genes in this process.

## 1. Introduction

Chronic myeloid leukemia (CML) is a clonal hematopoietic stem cell malignancy characterized by the presence of the chromosomal translocation t(9;22) generating Philadelphia (Ph1) chromosome in all leukemic cells [1]. The molecular counterpart the Ph1 chromosome is the chimeric *BCR-ABL* gene, which is translated in leukemic cells, into a constitutively active tyrosine kinase, which activates several signaling pathways. BCR-ABL is the driver oncogene at the origin of CML, as has been shown in animal models [2].

The natural history of the disease has been modified since the introduction of the tyrosine kinase inhibitors (TKIs), which became currently standard CML therapies as they induce deep molecular responses (DMR) translating into a major benefit in the survival of patients. However, several TKI discontinuation trials in the world have shown that even after several years of DMR, 50–60% of patients rapidly relapse, requiring to resume the treatment. The second major problem is the occurrence of resistance to TKI, leading to progression of the disease towards more aggressive phases [3]. A major issue remaining today in the CML field is the persistence of stem cells event in deep molecular response due to the resistance of leukemic stem cells to TKI currently in use [4,5]. This could be associated with clinical resistance in the context of TKI therapies with emergence of resistant clones from the initial CML stem cell clone, which has an inherent predisposition to genetic instability. In rare CML cases, progression to blast crisis still occurs, especially in this context, and the genomic events potentially predictive for progression are largely undetermined. The late events that accompany the progression towards blast crisis (BC) have been described and include an increase of BCR-ABL expression. Key molecules essential for the maintenance of stemness are shared by hematopoietic stem cells (HSCs) and leukemic stem cells (LSCs), including those implicated in transcription, regulation of cell cycle, metabolism, autophagy, signal transduction, and niche-associated factors. Wnt β-catenin signaling has been described in normal and pathological hematopoiesis including in CML [6,7]. Interestingly, a protein network including mainly MYC and p53 hubs has been shown to be associated with the LSC phenotype in the context of therapy escape during CML [8]. In this work, we characterized a stem cell program activated during CML blast crisis shared between chromatin binding of TCF7L2 and MYC. This transcriptional program defined to be independent of the adult hematopoietic program driven by RUNX1 and GATA2 and included epigenetics actors, which mainly implicated arginine methylation mechanism. Some TCF7L2 cis distal active enhancer like elements were also found near genomic regions of transcription factors activated during blast crisis.

## 2. Results

### 2.1. TCF7L2 Expression is Positively Correlated to the Number of Blast Cells in CML-BC

Transcriptome experiments performed on CD34+ cells from CML patients (GSE4170) were analyzed only for samples that were known to be in BC. The information about the peripheral blood blast cell numbers was available in 32 patients in BC, and four of these patients had therapy-induced remission. Blast numbers could be an important parameter reflecting the proliferative properties of the leukemic cells. The Pavlidis Template Matching (PTM) algorithm allowed to identify genes in the transcriptome positively correlated to the blast numbers (logarithm base 10). Ten percent of the transcriptome (1851 genes on a total of 18411 tested genes) was found to be positively correlated to the blast numbers (transformed in logarithm base 10) during the CML blast crisis (Supplementary Table S1). Twenty-five best-correlated genes with TCF7L2 allowed to discriminate by unsupervised classification BC samples from samples with remission (Euclidean distances and average method, Supplementary Figure S1).

**Figure 1:**
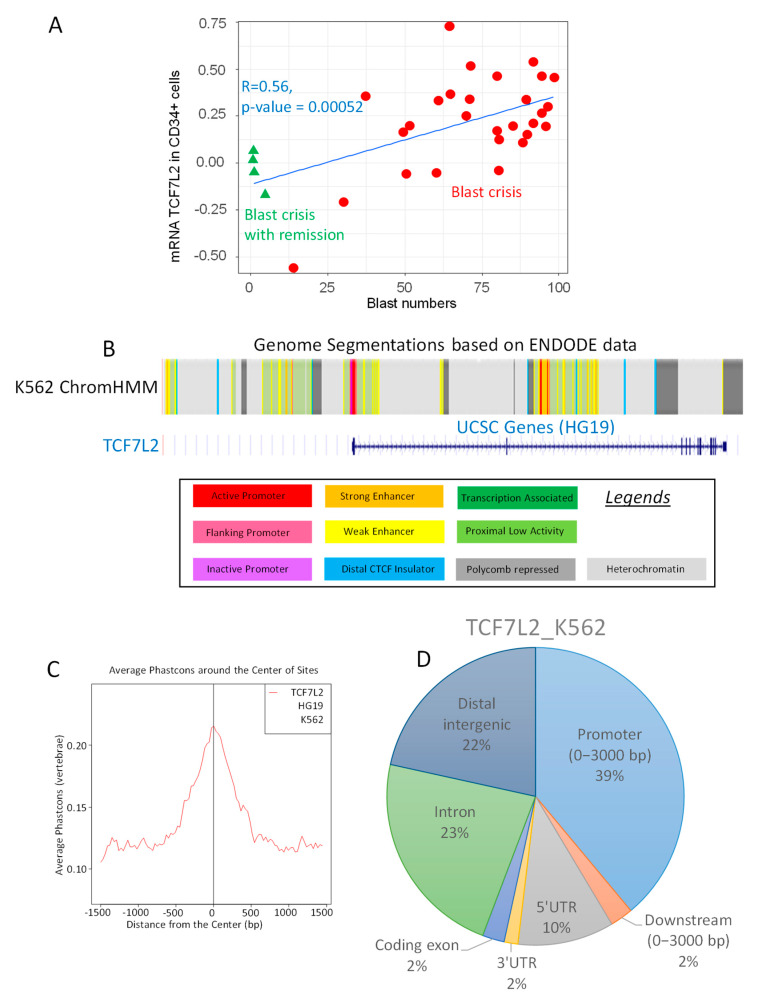
Regulation of TCF7L2 expression during CML blast crisis. (**A**) Scatterplot of TCF7L2 expression in CD34+ cells from patients in CML Blast Crisis versus the number of circulating blasts: red circles represent patients in blast crisis and green triangles represent patients in blast crisis with remission, linear model is presented as blue lines and results of the two-sided Pearson Correlation test is written in blue; (**B**) Functional Genomic Hidden Markov Model covering eight histone marks and CTCF chromatin binding for the activity of the TCF7L2 promoter in the K562 cell line; (**C**) Phast-conservation plot around Centers of the Sites for TCF7L2 chromatin binding by CHIP-sequencing (ENCODE data) in K562 normalized on vertebrae promoter database; (**D**) Pie chart of peak proportion in genomic regions concerning TCF7L2 chromatin binding in K562 cells and normalized on the HG19 version of the human genome.

Among the genes correlating with BC, the transcription factor TCF7L2, known as Transcription Factor 7 like 2 also known as alias Transcription Factor 4 (TCF4), was found to have a significant correlation to the blast cell numbers (Pearson correlation coefficient *r* = 0.52, two-sided Pearson test *p*-value = 0.00052, Figure 1A). Functional genomic analysis performed with ChromHMM algorithm on K562 cell highlighted an active promoter segment near Transcription Starting Site of TCF7L2 (Figure 1B). Epigenetic analysis of TCF7L2 CHIP-sequencing experiment performed on K562 (ENCODE consortium) revealed that binding events were found to be conserved around Transcription Starting Sites (TSS) in vertebrae promoter database (Figure 1C). More than one third of these events (39 percent) were found mapped in proximal promoter region on HG19 version of human genome (upstream 3000 pb, Figure 1D). Taking in account these results, it could be suggested that TCF7L2 could be a potential transcription factor active in the CML-BC.

### 2.2. TCF7L2 Transcriptional Program is Active during CML Blast Crisis

**Figure 2:**
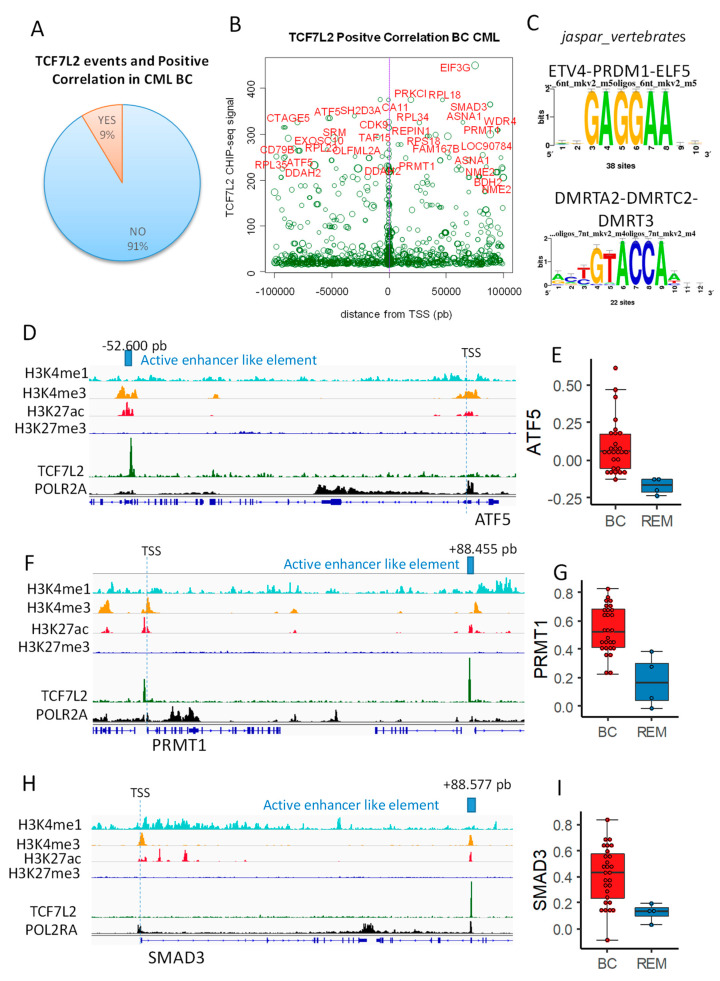
TCF7L2 epigenetic program allowed the identification of cis distal active enhancer like elements active during CML blast crisis: (**A**) pie chart showing proportion of TCF7L2 DNA binding event that was found with blast count positive correlation during CML BC; (**B**) Scatterplot TCF7L2 CHIPseq signal versus its position around TSS (+/−100 kb); the size of the circle events is proportional R Pearson Coefficient found between the gene and blast count during CML BC, genes with CHIP-seq signal over 200 are highlighted in red; (**C**) motif discovery found in the best TCF7L2 CHIP-seq peak (signal over 200, database: Jaspar core vertebrae 2020); (**D**) mapping of active enhancer like element found downstream ATF5; (**E**) ATF5 expression boxplot for patients in CML blast crisis (BC) as compared to patients in remission (REM); (**F**) mapping of active enhancer like elements found upstream PRMT1; (**G**) PRMT1 expression boxplot for patients in CML blast crisis (BC) as compared to patients in remission (REM); (**H**) mapping of active enhancer like element found upstream SMAD3; (**I**) SMAD3 expression boxplot for patients in CML blast crisis (BC) as compared to patients in remission (REM).

Previously, we found that 1851 genes were correlated to the blast number during the CML blast crisis (Supplementary Table S1). Complete analysis of distal and proximal TCF7L2 chromatin events (100 kb upstream and downstream around Transcription Starting Sites) revealed that nine percent of these events matched with promoter of genes which were found to be positively correlated with blast count during CML blast crisis (Figure 2A). Scatterplot of TCF7L2 signal mapped around TSS (+/−100kb: distal and proximal) showed that most important signal found (signal over 200 in CHIP-seq) is well distributed in upstream and downstream distal regions (Figure 2B). Additionally, this analysis allowed to observe maximum concentration of events around TSS in proximal region (near purple vertical line, Figure 2B). Thus, distal and proximal regions are both important to be analyzed. Among the most important was the TCF7L2 signal over 200 in CHIP-seq; 54 unique peaks were found after region mapping +/−100 kb around TSS, which correspond to 72 promoters of genes activated during CML blast crisis (Supplementary Table S2). These DNA binding event allowed enrichment of two redondant motifs, which are not compatible with TCF classical motifs (Figure 2C). In this distal analysis, some cis distal events were found to match with promoters mapped for some transcription factors, such as ATF5, SMAD3, PBX4, NKRF, REPIN1, and PRR3, but also with promoters of genes in implicated in stem cell signature, such as PRMT1 and NME2 (Supplementary Table S2 and Figure 2B). Cis Distal active enhancer like elements are distal regions active for polymerase 2 and regions are defined as genomic regions with special histone marks, comprising priming with H3K4me1 and H3K4me3 and H3K27Ac in absence of repressive mark H3K27me3 [9]. This characterization was performed for two transcription factors (ATF5 and SMAD3) and one stem cell marker (PRMT1) found in TCF7L2 distal program and activated during CML blast crisis (Figure 2D–I) and harboring important TCF7L2 CHIP-seq signal (Supplementary Table S2). These results suggest that TCF7L2 could interfere at genomic distal level to modified activation of transcription factors and stem molecules, which are important for the transcriptional program of the blast during CML progression disease.

**Figure 3:**
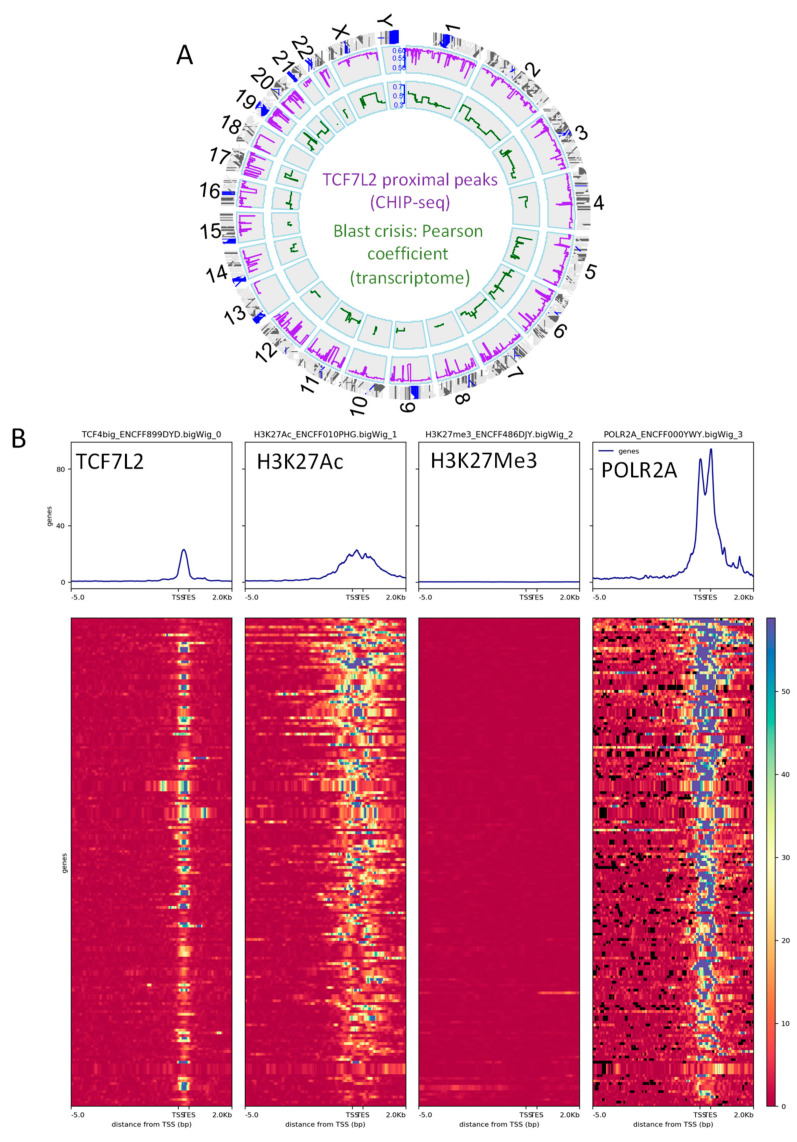
The integrative program of TCF7L2 in CML-BC is active for transcription: (**A**) Circos plot of the genomic landscape integrative program of TCF7L2 during the blast crisis of CML patients: the purple line represents the BETA algorithm score of proximal peaks positive for TCF7L2 in K562 cell line promoters (ENCODE data), the green line represent Pearson correlation coefficient of genes in CD34+ cells found to be correlated to the number of blasts in CML patients in BC, and the red gene symbol represent gene loci positive for the integrative analysis; (**B**) Promoter heatmap of the TCF7L2 genomic landscape positive in blast crisis of CML patients: promoter matrix was performed compared to histone mark H3K27Ac (active promoter mark) and POLR2A (positive for transcription), proximal signals were mapped from 5000 pb upstream and 2000 pb downstream from the Transcription Starting Sites (TSS).

Integration of the epigenetic binding program from TCF7L2 in transcriptome of CML blast crisis highlighted 183 proximal peaks (proximal −3000 pb upstream, +500 pb around TSS) among 1760 total proximal peaks, which correspond to the 144 genes active in the transcriptome during blast crisis. These TCF7L2 active targets were found to be well distributed all over in the human genome, except on chromosome 18 and chromosome Y (Figure 3A, Supplementary Table S3).

Functional epigenetics analyses revealed that the proximal promoter heatmap for TCF7L2 active targets during the blast crisis harbored an intensive signal around TSS for the RNA polymerase 2A, suggesting that these targets are active for transcription (Figure 3B). Additionally, these TCF7L2 targets harbored an intensive signal for histone H3 acetylation in proximal regions of the promoters (Figure 2B) and these same targets revealed that their promoters were negative for repressive epigenetic marks Histone-H3 (Figure 3B).

### 2.3. TCF7L2 Program Activated in CML-BC Shared with MYC Binding on Chromatin

**Figure 4:**
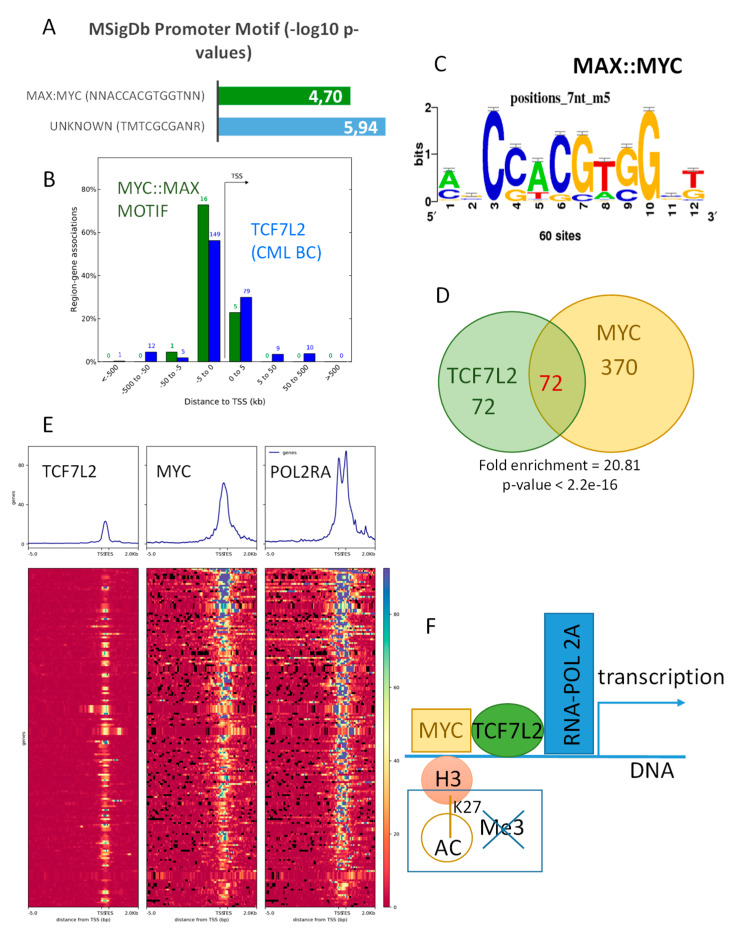
TCF7L2 shares half of its targets with MYC in CML-BC integrative program: (**A**) Bar plot of the promoter Motif enrichment performed on TCF7L2 genomic intervals correlated during blast crisis; results are presented as negative logarithm base 10 of *p*-values; (**B**) Histogram of regions around the Transcription Starting Sites concerning genomic intervals of TCF7L2 program during the blast crisis that are also positive for MYC::MAX motif binding sites (in blue, the totality of TCF7L2 program during the blast crisis, and in green, the sub-program positive for MYC::MAX binding sites); (**C**) MYC::MAX logo motif detection inside the TCF7L2 integrative program of the CML Blast Crisis; (**D**) Venn diagram comparing TCF7L2 and MYC genomic integrative programs, *p*-value of TCF7L2 program enrichment inside MYC ones was calculated by the Fisher hypergeometric test; (**E**) Promoter heatmap of the TCF7L2 genomic landscape positive in blast crisis of CML patients: promoter matrix was performed compared to MYC CHIP-sequencing (ENCODE data K562) and POLR2A (positive for transcription), proximal signals were mapped from 5000 pb upstream and 2000 pb downstream from the Transcription Starting Sites (TSS); (**F**) Schematic drawing summarizing the TCF7L2 binding mechanism on promoters activated during the blast crisis of CML, taking account of MYC interaction.

Functional enrichment performed with the GREAT algorithm on the TCF7L2 active program during the CML blast crisis revealed a significant enrichment with motif of transcription factor interaction MYC::MAX (*p*-value=1.15 × 10^−6;^ Figure 4A).

Mapping of this shared active program regulated by TCF7L2 and MYC/MAX interactions was found to be distributed around TSS of the promoters of the genes (Figure 4B). Analyzing MYC CHIP-sequencing performed on K562 (ENCODE project) showed that chromatin binding signal of this experiment is well centered and conserved around TSS of promoter database in vertebrae (Supplementary Figure S2A), and one third of these events were localized in proximal regions of the promoters (34 percent, Supplementary Figure S2B). Comparison of MYC epigenetics program with the previously identified TCF7L2 program during blast crisis highlighted a significant overlap (fold enrichment = 20.81, Fisher Exact test *p*-value < 2.2 × 10^−16^, Figure 4C,D). Effectively, half of the blastic program of TCF7L2 was found to be shared with the binding of MYC in proximal region of the promoters. The promoters of these shared programs between MYC and TCF7L2 were found positive for MYC and confirmed to be active for transcription because of the positivity for binding of RNA polymerase 2A (POLR2A) in proximal regions around TSS (Figure 4E). These results suggest that there exists an active transcriptional program during CML blast crisis, which is regulated by the shared binding of MYC and TCF7L2 in proximal regions of the gene promoters (Figure 4F).

### 2.4. Transcriptional Program Shared between TCF7L2 and MYC during Blast Crisis is Independent of the Hematopoietic Profile

**Figure 5:**
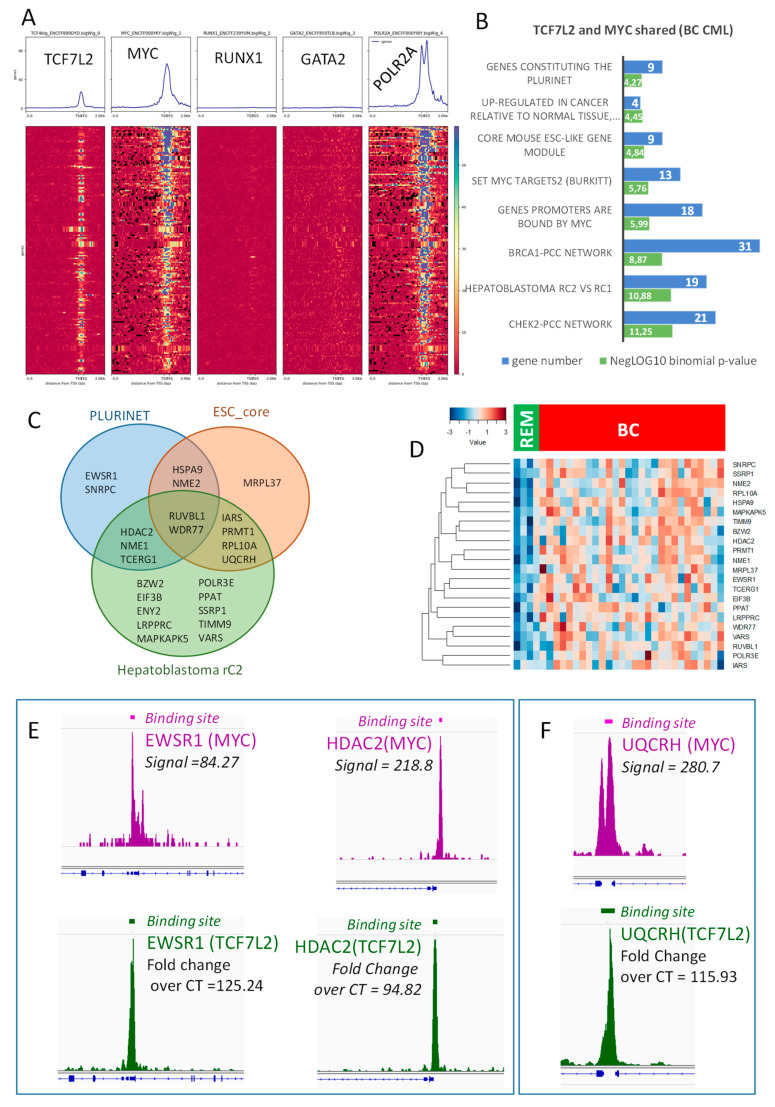
The TCF7L2 and MYC transcriptional program activated during blast crisis is independent of RUNX1/GATA2: (**A**) Promoter heatmap of shared TCF7L2-MYC genomic landscape positive in blast crisis of CML patients: the promoter matrix was performed compared to GATA2 and RUNX1 hematopoietic transcription factors CHIP-sequencing (ENCODE data K562) and POLR2A (positive for transcription), proximal signals were mapped from 5000 pb upstream and 2000 pb downstream from the Transcription Starting Sites (TSS); (**B**) Functional enrichment on MSigDb of the shared TCF7L2/MYC transcriptional program during blast crisis, bar plots represent the negative log10 of the binomial *p*-values and the number of genes implicated in each enriched functions; (**C**) Venn diagram highlighting stem cell enrichment in the blast crisis program shared by TCF7L2 and MYC; (**D**) Expression heatmap of stem cell related molecules found in transcriptional program shared by TCF7L2 and MYC during blast crisis of CML (BC: blast crisis, REM: blast crisis in remission); (**E**) CHIP-seq signal peak and binding site bars respective for MYC (purple) and TCF7L2 (green) concerning principal genes which overlap PLURINET signature; (**F**) CHIP-seq signal peak and binding site bars respective for MYC (purple) and TCF7L2 (green) concerning UQCRH, which overlaps the ESC core signature.

CML-associated transcriptional program allowed the identification of a shared binding for MYC and TCF7L2 active during blast crisis. In this program, we were able to test the potential involvement of hematopoietic transcription factors such as GATA2 and RUNX1. Surprisingly, this blastic program was found to be totally negative for the binding of these two hematopoietic transcription factors on the promoter heatmap presenting proximal CHIP-sequencing signals (Figure 5A).

Functional enrichment of this blastic program performed on MSigDb confirmed a significant enrichment for the myc targets (Figure 5B), but also with some enrichments in stem cell functionalities, such as the signature of C2 aggressive sub-group of hepathoblastoma, and signatures of core mouse Embryonic Cell and PLURINET network of stem cell genes (Figure 5B). These three stem cell phenotypes shared some regulated molecules that were highlighted on the following Venn diagram (Figure 5C) and present a higher level of expression in blast cells as compared to the cells during blast crisis in remission (Figure 5D). Among these molecules (Supplementary Table S4), some were found central in Venn diagram analysis, such as RUVBL1, which is a DNA helicase, and WDR77, alias MEP50, which is a partner of the arginine methyl transferase PRMT5. Another arginine methyltransferase (PRMT1) was found to be shared between core mouse embryonic profile and C2 hepatoblastoma signature (Figure 5C). Another epigenetic molecule HDAC2 (Histone Deacetylase 2) was found shared in these stem cell-regulated signatures. Two Nucleoside Diphosphate Kinases NME1 and NME2 were also found (Figure 5C,D) centrally implicated. These results suggest that the cooperative blastic program regulated by MYC/TCF7L2, implicating some stem cell molecules is independent of the classical hematopoietic program (GATA2 and RUNX1). Amongst the genes found to be activated during BC, some were found to overlap the PLURINET and ESC_core signature and also harbored important signals (proximal regions) in both MYC and TCF7L2 CHIP-sequencing experiments. This is the case for EWRS1 and HDAC2 concerning PLURINET signature (Figure 5E) and UQCRH concerning the ESC-core signature (Figure 5F). For others genes found to overlap PLURINET and ESC-core signatures, epigenetics peaks are presented in Supplementary Figure S3: they presented an important peak signal for MYC DNA-binding, and a weaker one for TCF7L2 in proximal regions of their promoter.

### 2.5. TCF7L2/MYC Cooperation is a Druggable Target

**Figure 6:**
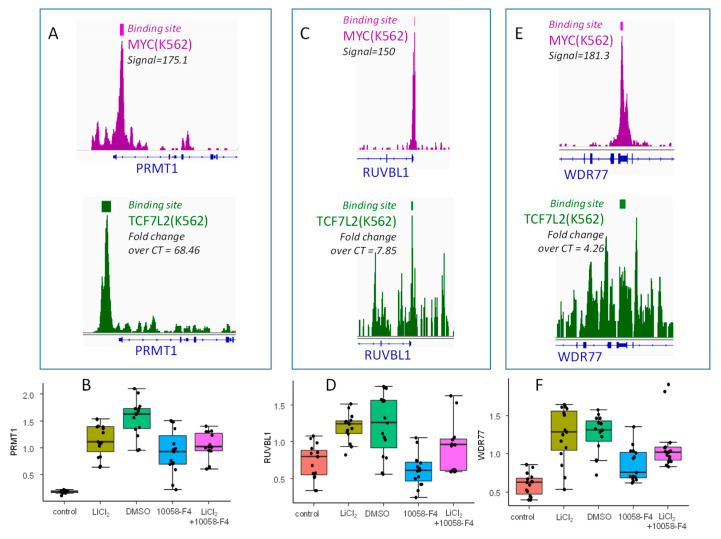
Validation of β-catenin and MYC dependent regulation of three embryonic related targets activated during CML blast crisis: (**A**,**C**,**E**) Respective Proximal CHIP-seq signal with binding site bars for MYC and TCF7L2 concerning PRMT1,RUVBL1, WDR77; (**B**,**D**,**F**) boxplots of respective QRT-PCR expression for PRMT1, RUVBL1, and WDR77 mRNA after stimulation of BCR-ABL positive hematopoietic progenitors (K562) with LiCl_2_ (control as control condition) and with 10058-F4 myc inhibitor (DMSO as control) and combination of the two stimulations, mRNA expression were normalized on GAPDH transcript quantification.

In order to confirm the involvement of the MYC and WNT/β-catenin pathways during a blast crisis, functional assays were performed using the K562 cell line. In vitro, β-catenin signaling activation via GSK3B was targeting by LiCl_2_ and MYC-MAX interaction was inhibited by 10058-F4 in order to confirm the cooperation of TCF7L2 and MYC to drive an embryonic program in the blast crisis of CML, especially by regulating some genes that were found to overlap PLURINET and ESCcore signatures: PRMT1, RUVBL1, and WDR77. PRMT1, RUVBL1, and WDR77 harbored important proximal signal in MYC CHIP-sequencing experiments (in purple, Figure 6A,C,E, respectively). PRMT1 was confirmed to present an important proximal signal for TCF7L2 CHIP-seq experiment (in green, Figure 6A), but this signal was found weaker for RUVBL1 and WDR77 (in green, Figure 6C,E, respectively). After in vitro stimulation without any cytotoxicity evaluated by trypan blue (Supplementary Figure S4), by QRTPCR analyses, some regulations were confirmed: PRMT1 (LiCl_2_: fold change = +6.33, *p*-value=8.49 × 10^−13^; 10058-F4: fold change =−1.69, *p*-value=6.52 × 10^−5^, n = 15, Figure 6B), RUVBL1 (LiCl_2_: fold change = +1.66, *p*-value=1.67 × 10^−6^; 10058-F4: fold change =−2.01, *p*-value=2.71 × 10^−5^, n = 15, Figure 6D), and WDR77 (LiCl_2_: fold change = +2.07, *p*-value=4.97 × 10^−7^; 10058-F4: fold change =−2.01, *p*-value=2.71 × 10^−5^, n = 15, Figure 6F). For these three genes, concomitant stimulation with the two treatments suggests an intermediary quantification found between the two individual treatments (Figure 6B,D,F). These results confirmed the regulation of blast crisis stem cell signature by cooperation of MYC and WNT/β-catenin pathways in Bcr-Abl positive cells; these actors are principally molecules implicated in transfer of methylation on arginine. Especially PRMT1, which presented a more deep regulation by LiCl_2_ stimulation, and its promoter presented a more important TCF7L2 proximal signal by CHIP-sequencing.

## 3. Discussion

BCR-ABL exhibits constitutive tyrosine kinase activity resulting in the development of leukemia through the stimulation of proliferative signaling pathways in the myeloid lineage. In this work, we characterized a transcriptional program activated during CML blast crisis shared between chromatin binding of TCF7L2 and MYC. It has been previously shown that BCR-ABL induces MYC expression [10]. MYC with TP53 was identified as a network hub in proteomic of hematopoietic progenitors from CML patients [8], and MYC has been demonstrated to be implicated in CML therapy resistance [11]. WNT/β-catenin signaling has also been shown to be implicated in normal and pathological hematopoiesis including CML [6,7]. A Link between TCF7L2, WNT/β-catenin and MYC pathways has been described during cardiac remodeling in heart failure [12] or in the context of pancreatic beta cell proliferation [13].

Previous work performed at single cell level in CML pathophysiology showed that a distinct signature could be highlighted during the progression of the disease with sub clonal expansion [14].

TCF7L2 Transcription Factor 7-like 2 alias Transcription Factor 4 (TCF4) is a high mobility group (HMG) box-containing transcription factor that could be activate after β-catenin accumulation [15]. In epigenetic experiments performed on colorectal cells, most TCF4-binding sites are located at large distances from transcription start sites [16]. Cis distal events of active element such as enhancers in a CML blast crisis transcriptional program could be important to investigate for this reason. Especially the TCF7L2 epigenetic program was found to merge with distal intervals compatible with active enhancers genomic segments near some transcription factors, such as ATF5, which has already been shown to be implicated in the regulation of autophagy of CML cells via the regulation of mTOR downstream BCR-ABL [17]. The TCF7L2 cis distal active enhancer genomic segment was also found at proximity of SMAD3 transcription factor. Smad3, a downstream effector of TGF-β signaling, is a “master regulator” of cell fate and could potentially act in the maintenance of “stemness” in CML cells [18].

Epigenetics of adult hematopoietic programs have previously been shown to be orchestrated by 10 major transcriptional regulators, including TAL1, LYL1, LMO2, GATA2, RUNX1, MEIS1, PU.1, ERG, FLI1, and GFI1B [19]. Runx1 (+/−)/Gata2 (+/−)heterozygous mice are not viable with severe hematopoietic defects at midgestation [19].

Mutations of Runx1 and Gata2 [20,21] have been described during CML-BC suggesting their major role in the control of the hematopoietic program during this phase. The highlighted cooperative role of TCF7L2 and MYC found in stem cell transcriptional program seems to be independent of the adult hematopoietic program driven by RUNX1 and GATA2 at chromatin binding level. This hematopoietic independence suggests that the program that we observed driven by TCF7L2/MYC cooperation profoundly reprograms leukemic cells during advanced phase CML and regulates a stem cell program involving pluripotent stem cell functionalities [22] shared also with an aggressive undifferentiated sub-group of hepatoblastoma [23]. TCF7L2/MYC program activated in this context of BC also involves epigenetics actors that mainly implicate the arginine methylation. Independently of the methyl transfer on arginine mechanism, in the blast crisis program, we identified HDAC2, a histone deacetylase. HDAC2 is implicated in epigenetics and have been shown to be associated to CFTR expression for PTEN-pathway regulation in Ph+ leukemia cell lines [24]. Interestingly, the PRMT1 gene, which promotes methyl transfers on arginine, is an unknown marker in CML pathophysiology and it was found to be implicated in the blast crisis stem cell program promoted by TCF7L2/MYC cooperation. PRMT1 has been identified as a key common downstream mediator for β-catenin/Hoxa9 functions in MLL in the context of cancer stem cells [25]. PRMT1 has already been proposed to be a potential epigenetic target in acute myeloid leukemia because it has been recognized to contribute to an aberrant epigenetic networks with KDM4C [26].

The CML-BC program that we describe also included the WD repeat protein (WDR77 gene, alias MEP50), which is a methylosome component. The complex arginine methyltransferase PRMT5-MEP50 is required for embryogenesis and is dysregulated in many cancers. PRMT5 targets a wide variety of substrates, including histone proteins involved in specifying an epigenetic code. MEP50 can interact with histones H2A and H4, which are known to be methylated by PRMT5 [27]. MEP50 binds PRMT5 and greatly enhances PRMT5’s histone methyltransferase ability. The PRMT5-MEP50 complex has a higher level of methyltransferase activity compared to PRMT5 alone [28]. MEP50 null mice are embryonic lethal, further supporting the essential function of the intact PRMT5-MEP50 complex [29,30]. Recently, it has been shown that targeting methyltransferase PRMT5 eliminates leukemia stem cells in chronic myelogenous leukemia [31]. Our work confirmed this mechanism by also highlighting the implication of MEP50 during blast crisis of CML to enhance methylation of arginine through PRMT5 activity. MEP50 can also interact with SUZ12, which is a Polycomb group protein that forms Polycomb repressive complexes (PRC2/3) together with EED and histone methyltransferase EZH2. SUZ12 might have a role in transcriptional regulation through physical interaction with MEP50 that can be an adaptor between PRMT5 and its substrate H2A [32]. PRMT5 methylation of histone H3R2 recruits Wdr5 a WD40-repeat protein and essential component of the MLL (mixed lineage leukemia lysine transferase) complex, stimulating H3K4 methylation and euchromatin maintenance [33]. Additionally, phosphorylation of MEP50 on T5 increases the methyltransferase activity of PRMT5-MEP50 toward histone H4, potentially by increased affinity for histone substrates [34]. MEP50 through is action on MEP50-PRMT50 could acts on Polycomb complex and potentially participates to epigenetic leukemia progression.

Stem cell program from a CML blast crisis contains molecules that could create a regulatory loop on myc/β-catenin oncogenic program. In this work, we demonstrated that RUVBL1, alias pontin, was regulated during BC by chromatin co-binding of MYC and TCF7L2 and it was confirmed by a functional assay in K562 cells. It is known that pontin associated with reptin has the ability to act on transcription through interactions with different transcription factors such as MYC and β-catenin-LEF/TCF and E2F to regulate proliferation and survival during tumorigenesis [35,36]. For example, in renal cell cancer, pontin knockout led to a decreased mRNA of both MYC and Cyclin D1, associated with a decrease of nuclear β-catenin expression [37]. Additionally, among the stem cell blast crisis program shared by MYC and TCF7L2, NME1 and NME2 are, respectively, Nucleoside Diphosphate Kinase 1 and 2, which play a major role in the synthesis of nucleoside triphosphates other than ATP. NME2 protein alias C-Myc Purine-Binding Transcription Factor PUF (nm23) acts as a transcriptional activator of the MYC gene [38]. Thus, these two molecules, RUVBL1 and NME2, found with TCF7L2 and MYC chromatin co-occupancy during CML blast crisis, could have a regulatory loop feedback on MYC and/or Wnt/β-catenin pathway. Pontin has also been demonstrated to be a critical regulator for AML1-ETO-induced leukemia via regulating the cell cycle progression in this model: pontin depletion inhibited leukemic self-renewal and caused cell cycle arrest [39].

**Figure 7:**
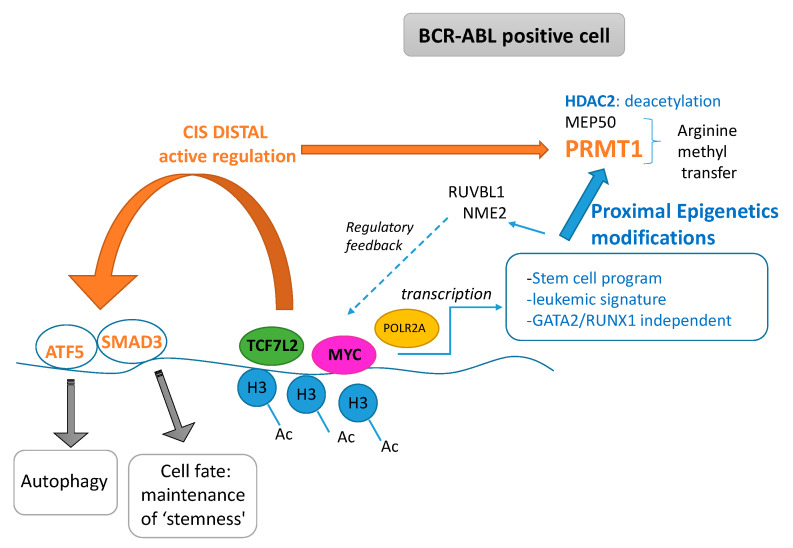
Pathophysiological model for cooperative role of TCF7L2 and MYC found in transcriptional program induced during CML blast crisis (Ac: acetyl, H3 histone H3).

In this work, we highlighted the cooperative role of TCF7L2 and MYC cooperation during the blast crisis of the CML to promote a transcriptional program which is independent of the hematopoietic transcriptional program (Figure 7). This program regulates molecules that have epigenetics functionalities such as deacetylation, and molecules implicated in the transfer of methyl on arginine. This program also linked RUVBL1 and NME2 to regulate the MYC and wnt/β-catenin pathways. Finally, the demonstration of the fact that this pathway can be targetable could open the way to future clinical applications. Some TCF7L2 cis distal active events could potentially activate important molecules implicated in stem cell biology.

## 4. Materials and Methods

### 4.1. Datasets Bioinformatics Integration

#### 4.1.1. Public Chip-sequencing Data

ENCODE consortium analyzed TCF7L2 binding sites in human cell lines K562 (Homo sapiens, adult 53 year female) by CHIP-sequencing [40] performed on an Illumina Genome Analyzer IIx. MYC, GATA2, RUNX2, and CHIP-sequencing in K562, also performed by ENCODE consortium, allowed us to investigate chromatin binding occupancy intersection with TCF7L2 CHIP-sequencing analysis. POLR2A (RNA polymerase2A) CHIP-sequencing in the K562 cell line allowed us to investigate active transcription site implicated in TCF7L2 chromatin binding occupancy analysis. Additionally, Histone marks H3K27Me3 and H3K27Ac allowed us to analyze the activity of promoters implicated in the TCF7L2 regulating program.

#### 4.1.2. Public Transcriptome Datasets

Transcriptome dataset of CD34+ cells from CML patients during progression of the disease (GSE4170) [41] was investigated to evaluate the regulation of the TCF7L2 and MYC target genes during blast crisis phase of CML according to blast count evaluation; normalized matrix of transcriptome Rosetta/Merck Human 25k v2.2.1 microarray was downloaded to be annotated with transcriptome annotation file number GPL2029.

### 4.2. Epigenetic Integrative Analyses

Bioinformatics analysis and graph representations were performed with R software environment version 3.2.3 by implementing several packages from Bioconductor and CRAN depositories: OmicCircos, Pheatmap, Factominer, ggplot2, made4. Transcriptome analysis matrix were firstly process with Multiexperiment viewer Mev version 4.9.0: Pavlidis Template Matching (PTM) algorithm [42] was applied on samples of patients in blast crisis and with the number of blasts (logarithm base 10 transformed) used as predictor. The BETA (Binding and Expression Target Analysis) algorithm was used to predict promoters affected by TCF7L2 binding in K562 cell lines [43]. Phast-Conservation plot performed on vertebrae promoter database was performed with python script form Tao Liu in Cistrome Galaxy pipeline. Genomics intervals predict by integrative analysis of TCF7L2 CHIP-sequencing integrated in Blast Crisis transcriptome were analyzed by the GREAT algorithm [44]. CHIP-sequencing promoter heatmap was established with the deeptools algorithm after computing matrix around reference region around Transcription Starting Sites: 5000 pb upstream and 2000 pb downstream [45]. CHIP-sequencing peak visualization was performed with Integrative Genomics Viewer version 2.3.40 standalone software [46].

### 4.3. Hematopoietic Cell Culture

K562 cell line was cultured in RPMI (Thermo Fisher, Waltham, Massachusetts) with 10% FCS. Th 10058-F4 inhibitor (Sigma Aldrich) was used at a concentration of 64 µM during 48 h, whereas LiCl_2_ (Sigma Aldrich, Saint Louis, Missouri) was used at a concentration 10 mM for 24 h. After incubation, viability and proliferation of the cells were evaluated with trypan blue (Supplementary Figure S4). Cells were then collected for RNA extraction.

### 4.4. RNA Extraction and Quantitative Reverse Transcription Polymerase Chain Reaction (QRT-PCR)

Total RNA from culture samples was performed with Trizol reagent by following the manufacturer’s instructions (Invitrogen, Carlsbad, CA) on five biological replicates. RNA quantification was performed with Nanodrop. Reverse transcription was performed starting with one microgram of total RNA in presence of random hexamer primers. cDNA was amplified in presence of SYBR green fluorescent reagent and couple of specific primers (Supplementary Table S5). Fluorescent signals (triplicates of PCR) were read in real time PCR on instrument MXPRO3005P Stratagene (Agilent Technologies, Santa Clara, CA). Ratio of relative expression for each transcript was determined after normalization on GAPDH housekeeping transcript with adapted formula 2^-deltaCT^ between target and housekeeping genes of each samples [47] PCR efficiency approximation to 100 percent was performed during these experiments.

### 4.5. Statistical Analysis

Statistical analysis was performed in R software environment version 3.4.3. Statistical significance between groups was assessed by performing two-sided Student t-test, correlation between variables was assessed by performing a two-sided Pearson correlation test, and a Fisher One Way Analysis of Variance (ANOVA) was used for multi-group comparison. For the tests, a *p*-value threshold of *p* < 0.05 was taken.

## Supplementary Materials

Supplementary materials can be found at https://www.mdpi.com/1422-0067/21/11/4057/s1. Supplementary Figure S1: Expression heatmap of 25 best correlated genes to blast number during blast crisis in the CML CD34+ progenitors; Supplementary Figure S2: MYC CHIP-seq analysis performed on K562 and provided by ENCODE consortium; Supplementary Figure S3: MYC and TCF7L2 CHIP-seq peak visualization for genes activated during the CML blast crisis and overlapping enrichment with PLURINET and ESC core signatures; Supplementary Figure S4: Boxplot presenting the ratios of dead cells/living cells after 48 h of culture and treatment stimulations of K562; Supplementary Table S1: table of genes that were found positively correlated with the number of blasts in CD34+ cells from CML patients in blast crisis phase; Supplementary Table S2: table of TCF7L2 distal and proximal best epigenetic events for genes candidates that were found positively correlated with the number of blasts in CD34+ cells from CML patients during blast crisis phase; Supplementary Table S3: table of proximal genomic intervals with promoter prediction for TCF7L2 program active during CML blast crisis; Supplementary Table S4: stem cell signature of the CML blast crisis promoted by TCF7L2/MYC chromatin proximal binding; Supplementary Table S5: Table of the QRT-PCR primers used in functional test to validate the regulation of TCF7L2-MYC targets.
