# Supplementary material for: Embryonic Program Activated during Blast Crisis of Chronic Myelogenous Leukemia (CML) Implicates a TCF7L2 and MYC Cooperative Chromatin Binding
Source: Int J Mol Sci. 2020 Jun 5;21(11):4057. doi: 10.3390/ijms21114057 (PMC7312032; doi:10.3390/ijms21114057)
Supplement: Supplementary file 1 [file ijms-21-04057-s001.pdf]

Embryonic Program Activated During Blast Crisis Of Chronic Myelogenous Leukemia (CML) Implicates A TCF7L2 and MYC Cooperative Chromatin Binding

Christophe Desterke[1,2], Patricia Hugues[1], Jin Wook Hwang[1], Annelise Bennaceur-Griscelli[1,2,3] and Ali G Turhan[1,2,3*]

[1] University Paris Saclay, INSERM U935, Villejuif, France;
[2] APHP Paris Saclay, University Paris Saclay, Villejuif, France
[3] INGESTEM Pluripotent Stem Cell Facility, Villejuif, France
* Correspondence: turviv33@gmail.com

Supplemental Material

Supplemental Figure

- **Supplemental Figure S1:** Expression heatmap of 25 best correlated genes to blast number during blast crisis in the CML CD34+ progenitors
- **Supplemental Figure S2:** MYC CHIP-seq analysis performed on K562 and provided by ENCODE consortium
- **Supplemental Figure S3:** MYC and TCF7L2 CHIP-seq peak visualization for genes activated during CML blast crisis and overlapping enrichment with PLURINET and ESC core signatures
- **Supplemental Figure S4:** Boxplot presenting ratios of dead cells/living cells after 48 hours of culture and treatment stimulations of K562

Supplemental Tables

- **Supplemental Table S1:** table genes which were found positively correlated with to the number of blasts in CD34+ cells from CML patients in blast crisis phase
- **Supplemental Table S2:** table of TCF7L2 distal and proximal best epigenetic events for genes candidates which were found positively correlated with the number of blasts in CD34+ cells from CML patients during blast crisis phase
- **Supplemental Table S3:** table of proximal genomic intervals with promoter prediction for TCF7L2 program active during CML blast crisis
- **Supplemental Table S4:** stem cell signature of the CML blast crisis promoted by TCF7L2/MYC chromatin proximal binding
- **Supplemental Table S5:** Table of the QRT-PCR primers used in functional test to validate the regulation of TCF7L2-MYC targets

Supplemental Figure

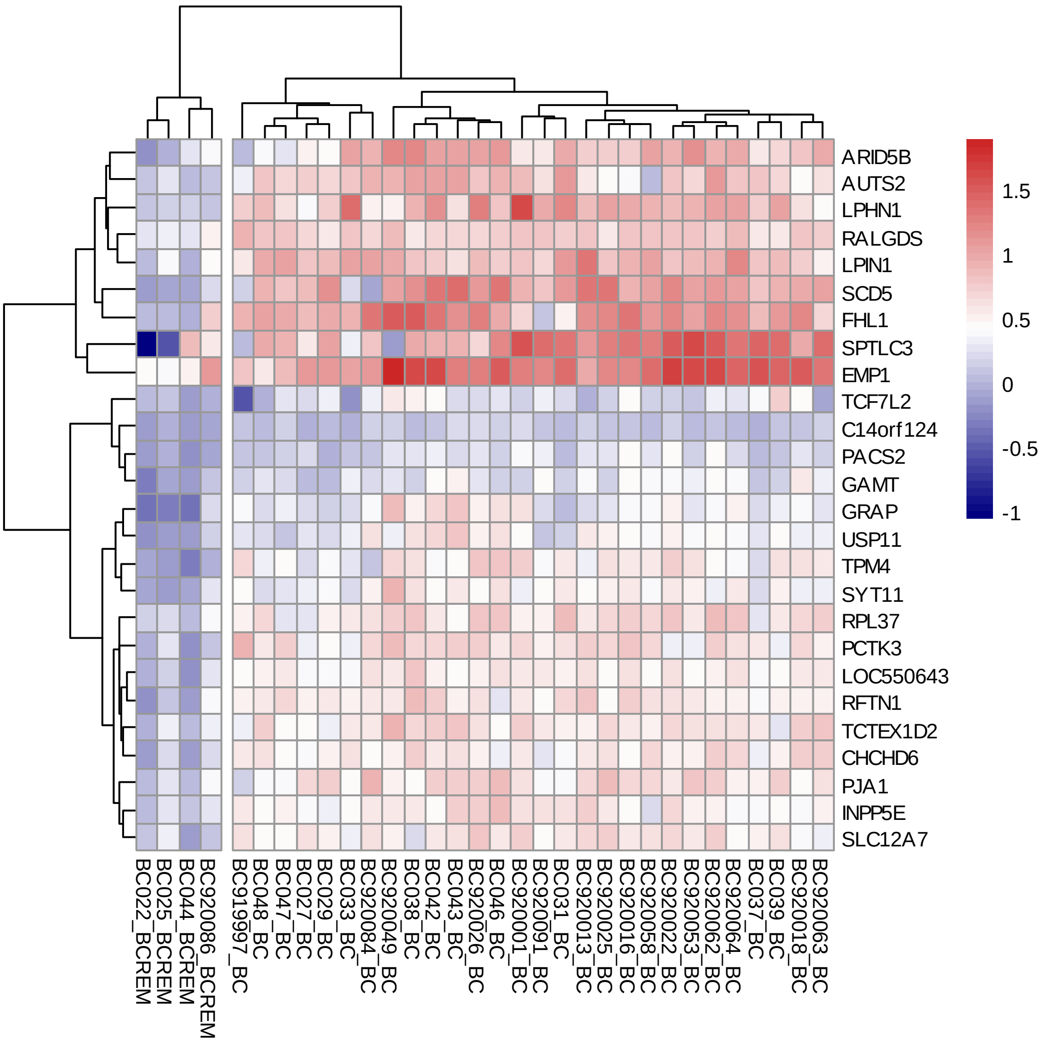

Supplemental Figure S1: Expression heatmap of 25 best correlated genes to blast number during blast crisis in the CML CD34+ progenitors: (Euclidean distances), sample identifiers: BC: CML Blast crisis, BC-REM: CML blast crisis in remission

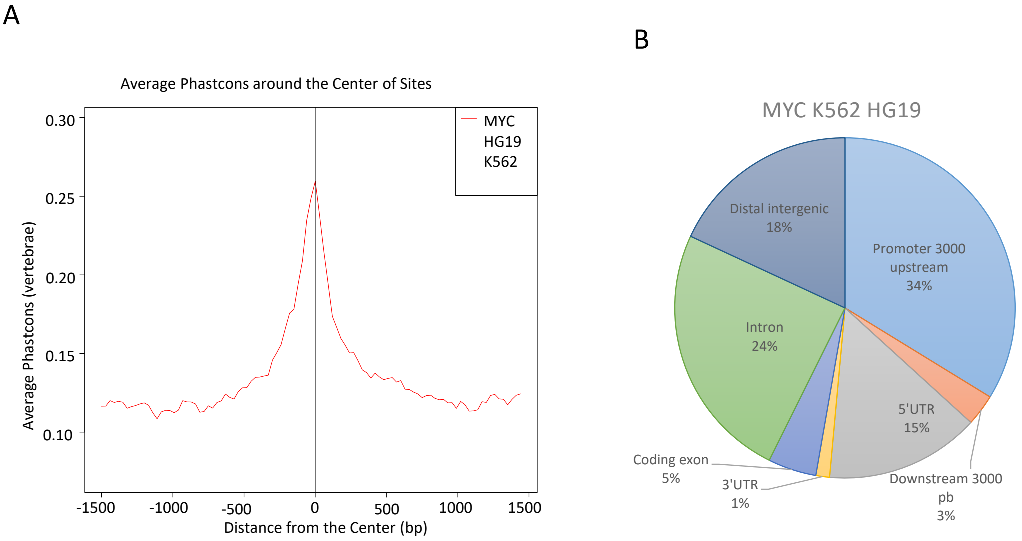

Supplemental Figure S2: MYC CHIP-seq analysis performed on K562 by ENCODE consortium: (**A**) Phast-conservation plot showing that signal of the CHIP-seq is centered and conserved (Mammalian evolution) around Transcription Starting Sites of the promoters; (**B**) Pie chart of TCF7L2 CHIP-seq signal proportion in different genomic areas after analysis on HG19 version of human genome.

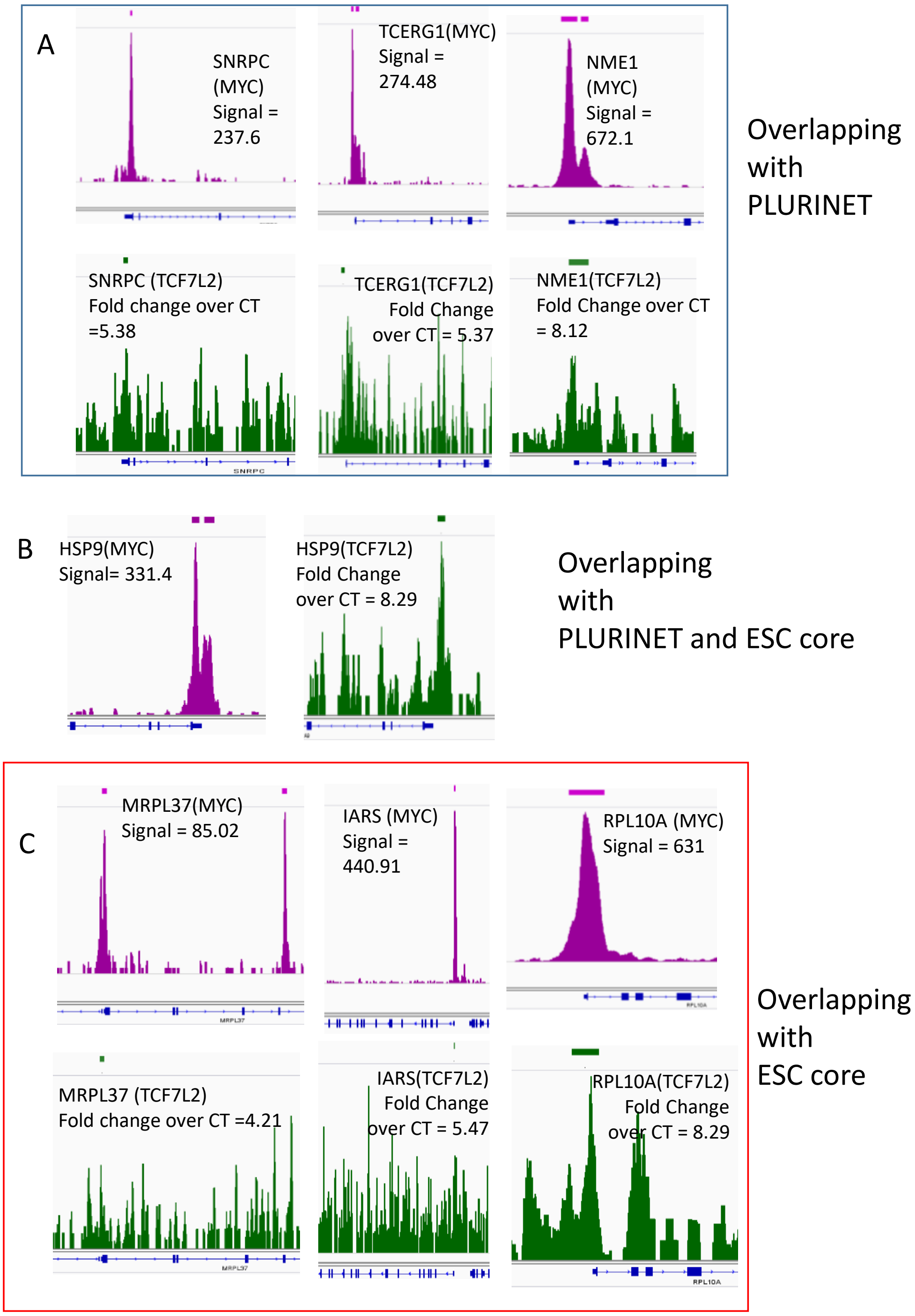

Supplemental Figure S3: MYC and TCF7L2 CHIP-seq peak visualization for genes activated during CML blast crisis and overlapping enrichment with PLURINET and ESC core signatures: (**A**) CHIP-SEQ peak visualization (MYC and TCF7L2) for activated genes during CML Blast crisis and overlaps with PLURINET signature; (**B**) CHIP-SEQ peak visualization (MYC and TCF7L2) for activated genes during CML Blast crisis and overlaps with PLURINET and ESC core signatures; (**C**) CHIP-SEQ peak visualization (MYC and TCF7L2) for activated genes during CML Blast crisis and overlaps with ESC core signature

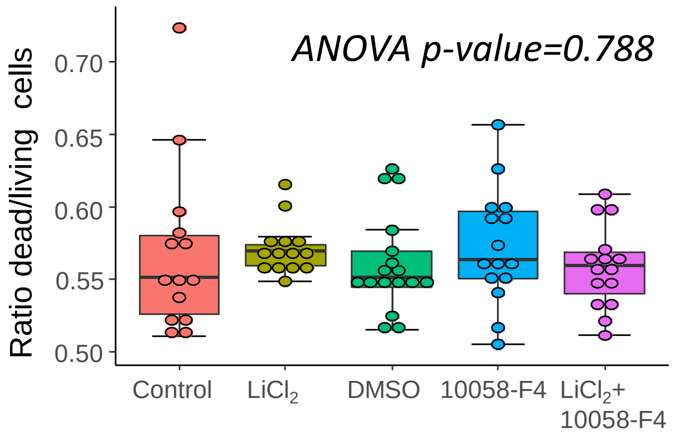

Supplemental Figure S4: Boxplot presenting ratios of dead cells/living cells after 48 hours of culture and treatment stimulations of K562: p-value between groups was estimate by Fisher One Way Analysis of Variance (ANOVA)

Supplemental Tables

Supplemental Table S1: table of genes which were found positively correlated with to the number of blasts in CD34+ cells from CML patients in blast crisis phase: Pavlidis Template Matching algorithm was processed on CD34+ cell transcriptome from Blast Crisis CML patients by using logarithm 10 of Blast number as predictor, table presents gene symbols with their gene identifier number with results from the algorithm with respective R Pearson Coefficient, raw p-values and False Discovery Rates corrected q values

Gene symbol	Pearson R values	Pearson p Values	FDR q Values
SCD5	0.79538894	0.0000000534	0.0000988434
LOC550643	0.7695273	0.000000264	0.0002443320
RFTN1	0.73851615	0.0000014	0.0007265175
TPM4	0.73613715	0.00000157	0.0007265175
ARID5B	0.7304172	0.00000208	0.0007700160
RPS11	0.725591	0.00000262	0.0008082700
SYT11	0.72213715	0.00000308	0.0008144400
RALGDS	0.7175979	0.00000379	0.0008720267
LPIN1	0.7151554	0.00000424	0.0008720267
GRAP	0.70720905	0.00000603	0.0011161530
SPTLC3	0.70316523	0.00000718	0.0011476200
TCTEX1D2	0.7005381	0.00000804	0.0011476200
C14orf124	0.69808626	0.00000892	0.0011476200
USP11	0.6963287	0.0000096	0.0011476200
AUTS2	0.69627076	0.00000962	0.0011476200
CHCHD6	0.6955417	0.00000992	0.0011476200
PACS2	0.6921637	0.0000114	0.0012412588
GAMT	0.6834278	0.0000162	0.0015733500

INPP5E	0.68244165	0.0000169	0.0015733500
PCTK3	0.6822166	0.000017	0.0015733500
RPL37	0.68057925	0.0000182	0.0016042000
FHL1	0.6785741	0.0000197	0.0016427625
LPHN1	0.6768655	0.000021	0.0016427625
SLC12A7	0.67656195	0.0000213	0.0016427625
PJA1	0.6749405	0.0000226	0.0016733040
EMP1	0.67354923	0.0000239	0.0017014962
AOF2	0.6697624	0.0000276	0.0018078100
YWHAE	0.668487	0.0000289	0.0018078100
PLXNB2	0.6681913	0.0000293	0.0018078100
CCT7	0.668141	0.0000293	0.0018078100
RAB15	0.6653177	0.0000326	0.0019465355
ZNF827	0.65982115	0.0000399	0.0022548545
CRY1	0.6595834	0.0000402	0.0022548545
DDAH2	0.65564245	0.0000464	0.0024294375
PRMT1	0.6539978	0.0000492	0.0024294375
SET	0.6538949	0.0000494	0.0024294375
VEGFB	0.6535766	0.0000499	0.0024294375
RPL13	0.65245926	0.0000519	0.0024294375
HKR1	0.6524357	0.000052	0.0024294375
PARP1	0.65214914	0.0000525	0.0024294375
LOC728705	0.64991623	0.0000568	0.0025409182
SLC41A3	0.64874846	0.0000592	0.0025409182
ARL6IP1	0.64837134	0.00006	0.0025409182
BIN1	0.64815456	0.0000604	0.0025409182
GPC1	0.6473452	0.0000621	0.0025543800
PTPRCAP	0.6464233	0.0000642	0.0025833522
AHDC1	0.64492977	0.0000676	0.0026437868
TSC22D3	0.643708	0.0000705	0.0026437868
STARD9	0.64363194	0.0000707	0.0026437868
RALGDS	0.643172	0.0000718	0.0026437868
NME1	0.642063	0.0000746	0.0026437868
ZNF251	0.64181507	0.0000752	0.0026437868
SRM	0.64160264	0.0000757	0.0026437868
BLMH	0.63720274	0.0000879	0.0030130167
DFFA	0.63606626	0.0000913	0.0030726600
DNPEP	0.63431054	0.0000968	0.0031954105
CSNK1E	0.63382196	0.0000984	0.0031954105
SDCCAG3	0.6314394	0.000106	0.0033828621
SCARB1	0.62930965	0.000114	0.0035765085
SEPTIN6	0.62884855	0.000116	0.0035786000
PTMA	0.6281697	0.000118	0.0035806230
RSL1D1	0.62765044	0.00012	0.0035825806
UCKL1	0.6269262	0.000123	0.0035863125

HCN3	0.62671	0.000124	0.0035863125
ATIC	0.62622434	0.000126	0.0035880923
ZNF256	0.6248229	0.000132	0.0036215217
MYL6B	0.62446254	0.000133	0.0036215217
PRKX	0.6243321	0.000134	0.0036215217
LOC401577	0.624196	0.000135	0.0036215217
WDR60	0.62372243	0.000137	0.0036226714
GNA15	0.6214961	0.000147	0.0038034247
PTPLA	0.6211901	0.000148	0.0038034247
LGTN	0.62089044	0.00015	0.0038034247
EHMT2	0.6196721	0.000155	0.0038770946
CCDC25	0.61888987	0.000159	0.0039183506
DDHD1	0.6184739	0.000161	0.0039183506
AKR1B1	0.6181871	0.000163	0.0039183506
GDF11	0.61283433	0.000192	0.0044641765
NAT9	0.61247337	0.000195	0.0044641765
TPM2	0.61184084	0.000198	0.0044641765
RABEP2	0.61179453	0.000199	0.0044641765
LRP8	0.6115719	0.0002	0.0044641765
C14orf112	0.61123854	0.000202	0.0044641765
MAP3K14	0.6111394	0.000203	0.0044641765
RBM15B	0.610761	0.000205	0.0044641765
CXXC5	0.60974085	0.000212	0.0045530345
KDSR	0.6094192	0.000214	0.0045530345
FLJ20628	0.60846126	0.00022	0.0046275000
PIK3IP1	0.607999	0.000223	0.0046378989
CROCC	0.6075834	0.000226	0.0046480667
UBE2L6	0.606564	0.000233	0.0047393736
RPL13A	0.605568	0.00024	0.0047966774
FSCN1	0.60542417	0.000241	0.0047966774
LAS1L	0.6033509	0.000257	0.0049745625
SIDT1	0.6032052	0.000258	0.0049745625
RPL23A	0.60315895	0.000258	0.0049745625
DST	0.60234016	0.000265	0.0050568557
A1BG	0.60193735	0.000268	0.0050619184
PRDX1	0.6013662	0.000272	0.0050855758
C12orf23	0.600776	0.000277	0.0051272700
IMPDH2	0.5984634	0.000297	0.0052959167
GPC3	0.59840935	0.000297	0.0052959167
DFFA	0.59828264	0.000298	0.0052959167
RALYL	0.5982637	0.000299	0.0052959167
PQBP1	0.5977373	0.000303	0.0052959167
ACP1	0.5974239	0.000306	0.0052959167
CD81	0.5972597	0.000307	0.0052959167
CDK6	0.59705913	0.000309	0.0052959167

TIGD7	0.595335	0.000325	0.0055190367
GPSM1	0.5949618	0.000329	0.0055361727
PRMT5	0.5943236	0.000335	0.0055863514
ESD	0.59314793	0.000346	0.0057182679
ADAM20	0.5926867	0.000351	0.0057495664
ZNF71	0.5922263	0.000356	0.0057803158
HS2ST1	0.5916114	0.000362	0.0058266261
LGALS9	0.5905698	0.000373	0.0059485128
EIF3G	0.59027606	0.000376	0.0059485128
POLD2	0.58978534	0.000382	0.0059922203
LRPPRC	0.58917826	0.000388	0.0060351933
KLC1	0.58828044	0.000398	0.0061391500
LGALS9	0.58725995	0.00041	0.0062193600
LAP3	0.5870696	0.000412	0.0062193600
NPM1	0.58658975	0.000418	0.0062193600
RAB39B	0.5865306	0.000419	0.0062193600
CAMTA1	0.5863837	0.00042	0.0062193600
TRAF4	0.5861226	0.000424	0.0062287619
DIMT1L	0.5854766	0.000431	0.0062574851
RPS18	0.58515626	0.000435	0.0062574851
HMGN3	0.5848234	0.000439	0.0062574851
GCN5L2	0.58460784	0.000442	0.0062574851
C21orf34	0.5840226	0.000449	0.0062574851
ARID5B	0.5839758	0.00045	0.0062574851
AVEN	0.5839339	0.00045	0.0062574851
STRBP	0.58369875	0.000453	0.0062574851
CYP2J2	0.582164	0.000473	0.0064853556
NOB1	0.5817628	0.000479	0.0065122774
ATXN10	0.58148193	0.000482	0.0065122774
WASF1	0.5810456	0.000488	0.0065455652
THSD1	0.57995063	0.000503	0.0066982230
TMSB10	0.5797091	0.000507	0.0067032643
SLC39A10	0.57869256	0.000521	0.0068395106
RCN1	0.5782084	0.000528	0.0068825915
ZMIZ1	0.57727414	0.000542	0.0070082690
ACP1	0.57680535	0.000549	0.0070082690
AEBP1	0.5768033	0.000549	0.0070082690
ULK3	0.5751686	0.000574	0.0072569007
HINT1	0.57453954	0.000584	0.0072569007
PDCL3	0.57451475	0.000584	0.0072569007
CCNB1IP1	0.5743574	0.000587	0.0072569007
PSMA1	0.57410306	0.000591	0.0072569007
ZNF598	0.57401973	0.000592	0.0072569007
DPH5	0.57341975	0.000602	0.0073309342
NOL14	0.5730536	0.000608	0.0073556078

EIF3M	0.5726697	0.000614	0.0073571392
DCTD	0.57233924	0.00062	0.0073571392
GNB5	0.5722148	0.000622	0.0073571392
EIF4A2	0.57196593	0.000626	0.0073571392
TCEA2	0.5718437	0.000628	0.0073571392
NUTF2	0.571363	0.000636	0.0074040000
RNF125	0.5696238	0.000667	0.0077163562
ABHD14B	0.56923515	0.000674	0.0077239259
DDX47	0.5691231	0.000676	0.0077239259
PWP1	0.56859285	0.000685	0.0077538841
GTF3A	0.5685267	0.000687	0.0077538841
CMTM3	0.5679773	0.000697	0.0078190727
ITPR1	0.5674769	0.000706	0.0078723253
DDEF2	0.56641555	0.000726	0.0080468623
CLCN6	0.56597495	0.000735	0.0080502071
CD52	0.56594986	0.000735	0.0080502071
ZBED3	0.5654453	0.000745	0.0080751228
ABI2	0.56540334	0.000746	0.0080751228
RRAS2	0.5648776	0.000756	0.0081357907
RPS5	0.5644013	0.000766	0.0081957572
RPL18A	0.564025	0.000774	0.0082337586
PSME2	0.563762	0.000779	0.0082395943
DDX51	0.5634245	0.000786	0.0082663977
FBXO32	0.56214434	0.000813	0.0084750843
C20orf4	0.56205577	0.000815	0.0084750843
CCT4	0.5615481	0.000826	0.0085414860
BANF1	0.56121314	0.000833	0.0085660167
UBC	0.5604482	0.00085	0.0086678548
POLR1C	0.5602979	0.000853	0.0086678548
SP2	0.56011844	0.000857	0.0086678548
EIF2S3	0.559515	0.00087	0.0086678548
C21orf57	0.5594781	0.000871	0.0086678548
NCBP2	0.5594672	0.000871	0.0086678548
MTMR2	0.55900776	0.000882	0.0087163492
C14orf179	0.5587878	0.000887	0.0087163492
ELP2	0.55863297	0.00089	0.0087163492
MDK	0.55823064	0.0009	0.0087219895
FAM60A	0.5582201	0.0009	0.0087219895
QPRT	0.5578746	0.000908	0.0087536875
TCF3	0.5572183	0.000924	0.0088617824
DRG2	0.556985	0.000929	0.0088638093
DCLK1	0.5562905	0.000946	0.0089797231
C1orf123	0.5559728	0.000954	0.0090094592
ZNF76	0.55547965	0.000966	0.0090399848
TRIM44	0.55543065	0.000967	0.0090399848

SEPTIN11	0.5552125	0.000972	0.0090410653
ANKRD13B	0.55481535	0.000982	0.0090884100
PLXNB3	0.5545048	0.00099	0.0091168657
CSNK1E	0.5539651	0.001003926	0.0091424717
CD69	0.55361736	0.001012862	0.0091424717
NMT2	0.5535374	0.001014926	0.0091424717
RNASEH1	0.5533559	0.001019628	0.0091424717
ETS2	0.55325335	0.001022292	0.0091424717
COPS8	0.55315715	0.001024797	0.0091424717
AGPAT4	0.5530591	0.001027355	0.0091424717
MCCC2	0.5522226	0.001049411	0.0092940658
GIMAP2	0.55157363	0.001066808	0.0094031505
DDX47	0.55022967	0.001103642	0.0096817125
CLSTN1	0.5493949	0.001127077	0.0098054422
CHIC1	0.5493503	0.001128341	0.0098054422
AK3	0.5486766	0.001147591	0.0099261259
RNF125	0.547995	0.001167359	0.0100501466
ACAA2	0.5474717	0.001182736	0.0101353904
RPS9	0.54688305	0.001200247	0.0102143105
ITPR1	0.5467918	0.001202982	0.0102143105
AIFM2	0.5458795	0.001230624	0.0103556922
E2F6	0.5456352	0.001238118	0.0103556922
RASSF8	0.54553735	0.001241132	0.0103556922
VDAC2	0.5452669	0.001249494	0.0103556922
CYFIP1	0.54520494	0.001251417	0.0103556922
CPXM1	0.54514754	0.001253201	0.0103556922
ITPR1	0.54463387	0.001269262	0.0104417954
ISG20L1	0.5439645	0.00129046	0.0105157492
TRMU	0.54385495	0.001293959	0.0105157492
IHPK2	0.5437538	0.001297197	0.0105157492
ZNF32	0.543636	0.001300976	0.0105157492
PRNPIP	0.543134	0.001317193	0.0105593920
LOC646778	0.5427968	0.001328183	0.0105593920
RBMX	0.5421203	0.001350477	0.0105593920
PBRM1	0.5420892	0.001351508	0.0105593920
APEX1	0.5420046	0.001354322	0.0105593920
C3orf37	0.54194194	0.001356408	0.0105593920
BDH2	0.54186225	0.001359066	0.0105593920
SRP72	0.5416174	0.00136726	0.0105593920
EXOSC10	0.5415882	0.00136824	0.0105593920
YWHAQ	0.54156286	0.001369091	0.0105593920
PPM1E	0.5415618	0.001369127	0.0105593920
GLO1	0.54125416	0.001379496	0.0105952162
PERLD1	0.54072374	0.001397535	0.0106592311
PRPS1	0.54067075	0.001399348	0.0106592311

C5orf42	0.54041666	0.001408073	0.0106776106
TARSL2	0.54026514	0.001413298	0.0106776106
GNL1	0.54002655	0.00142156	0.0106920055
BCAT1	0.5398771	0.001426756	0.0106920055
TRMT1	0.5395841	0.001436993	0.0107252986
ADA	0.53919655	0.001450631	0.0107836063
CIAPIN1	0.5383972	0.001479119	0.0109393805
INPP5F	0.53827804	0.001483406	0.0109393805
SOX4	0.5378817	0.001497749	0.0110013230
CRIM1	0.5374043	0.001515183	0.0110836714
TSPYL2	0.53724784	0.001520936	0.0110836714
NAT11	0.53701526	0.001529522	0.0111025303
EWSR1	0.5367986	0.001537559	0.0111172723
CDK6	0.5363417	0.001554627	0.0111969439
HSCB	0.5360921	0.001564023	0.0111973255
MUTYH	0.53590786	0.001570989	0.0111973255
LOC728769	0.53585935	0.001572828	0.0111973255
SH3PXD2B	0.53534675	0.001592372	0.0112930290
SHANK3	0.5343078	0.001632637	0.0115343935
SPR	0.53384805	0.001650735	0.0116179106
ARHGAP22	0.53349864	0.001664607	0.0116711650
PBXIP1	0.5332145	0.001675962	0.0117064365
ACTN1	0.53290516	0.001688402	0.0117222904
TSPAN3	0.5326697	0.001697924	0.0117222904
ANKMY1	0.53256804	0.00170205	0.0117222904
LOC728131///FLJ12993	0.53233683	0.001711466	0.0117222904
PANX1	0.5321745	0.001718103	0.0117222904
LARS2	0.5319716	0.001726434	0.0117222904
CUBN	0.5318287	0.00173232	0.0117222904
C9orf91	0.5317284	0.001736463	0.0117222904
DENR	0.53145826	0.001747662	0.0117222904
HSP90AB1	0.53141266	0.001749558	0.0117222904
KIAA0082	0.5313439	0.001752423	0.0117222904
TNS3	0.5312275	0.001757277	0.0117222904
COQ6	0.53093743	0.001769432	0.0117222904
LOC157503	0.53087634	0.001772002	0.0117222904
CD74	0.53084725	0.001773226	0.0117222904
PRPF19	0.5304615	0.001789533	0.0117483232
VDAC1	0.5304346	0.001790674	0.0117483232
ITGA5	0.53030455	0.001796205	0.0117483232
ZNF207	0.5299729	0.001810374	0.0117534228
NME2	0.52990484	0.001813295	0.0117534228
COX7B2	0.52984107	0.001816034	0.0117534228
ZNF22	0.52923954	0.001842055	0.0118396194
WDR54	0.52923745	0.001842145	0.0118396194

NDUFS3	0.5290868	0.001848712	0.0118407125
ERGIC1	0.5287913	0.001861654	0.0118824881
ZNF16	0.52858377	0.00187079	0.0118831728
GTF2H4	0.52849746	0.001874601	0.0118831728
AGPAT4	0.52813685	0.001890597	0.0119038817
LYRM4	0.52813387	0.00189073	0.0119038817
RPS4X	0.52766204	0.00191184	0.0119473574
GNB5	0.5276085	0.001914248	0.0119473574
ST3GAL3	0.5275474	0.001916999	0.0119473574
TRIAP1	0.52736145	0.001925395	0.0119594166
ALKBH3	0.5268848	0.001947062	0.0120292124
OVGP1	0.5267582	0.001952853	0.0120292124
PLEKHG2	0.5266867	0.001956128	0.0120292124
CTXN1	0.52635	0.001971624	0.0120521787
C14orf166	0.5263227	0.001972885	0.0120521787
IL3RA	0.52609867	0.001983261	0.0120757109
HHAT	0.5257764	0.001998269	0.0120760989
FAM116B	0.5257408	0.001999932	0.0120760989
TCF4	0.52567744	0.002002897	0.0120760989
GLTSCR2	0.525394	0.002016203	0.0121168563
SART1	0.5239984	0.002082858	0.0124093742
SF3B3	0.523526	0.002105848	0.0124093742
APOO	0.52346283	0.00210894	0.0124093742
MIRN155	0.52344245	0.002109938	0.0124093742
SSBP2	0.5232357	0.002120088	0.0124093742
UNK	0.5231356	0.002125016	0.0124093742
TOMM70A	0.5228089	0.002141171	0.0124093742
NAT14	0.52278715	0.002142251	0.0124093742
TAF15	0.52275586	0.002143804	0.0124093742
DISP1	0.5227463	0.002144278	0.0124093742
SETD1A	0.52252907	0.002155095	0.0124093742
PER1	0.52252805	0.002155145	0.0124093742
KCNA1	0.5225201	0.002155541	0.0124093742
SLC25A1	0.52245617	0.002158735	0.0124093742
ZNF84	0.5223184	0.002165627	0.0124104507
FAIM	0.5217953	0.002191979	0.0125226948
ADCY6	0.52090484	0.002237472	0.0127432636
NXT1	0.52052826	0.002256958	0.0128148137
RPL3L	0.52032924	0.002267315	0.0128342510
WDR74	0.5201356	0.002277433	0.0128522210
PFDN1	0.518541	0.002362247	0.0132896850
LNPEP	0.5183409	0.002373081	0.0132896850
GEMIN8	0.5180855	0.002386972	0.0132896850
STAP1	0.51790756	0.00239669	0.0132896850
ZDHHC21	0.5178605	0.002399268	0.0132896850

LOC387723	0.5176315	0.002411839	0.0132896850
MRPS35	0.51743525	0.002422656	0.0132896850
HIGD1A	0.51743126	0.002422876	0.0132896850
ITPR1	0.51734346	0.002427731	0.0132896850
CDK6	0.51716	0.002437901	0.0132896850
PAQR3	0.51693404	0.002450477	0.0132896850
USP13	0.5169136	0.002451618	0.0132896850
OSBPL3	0.5168147	0.002457142	0.0132896850
PHB2	0.51679903	0.002458019	0.0132896850
LOC441108	0.5165989	0.002469236	0.0132896850
RPAP1	0.51658833	0.002469828	0.0132896850
PHF16	0.5163426	0.002483664	0.0133072231
PRDX3	0.5162588	0.002488398	0.0133072231
PPM1J	0.5161482	0.002494655	0.0133072231
FAM167B	0.51600873	0.002502568	0.0133110729
MRPS30	0.51556754	0.002527742	0.0134064483
ERC2	0.51464224	0.002581252	0.0136511356
MAN1C1	0.5142657	0.002603306	0.0136768089
R3HDM1	0.5142489	0.002604294	0.0136768089
ATP5C1	0.51417685	0.002608535	0.0136768089
ZFP30	0.51405597	0.002615662	0.0136768089
RPL18	0.51381284	0.002630048	0.0137093535
NFATC2	0.51370084	0.002636699	0.0137093535
MRPS27	0.51331604	0.002659658	0.0137156741
ARL3	0.5132391	0.00266427	0.0137156741
FBXO32	0.5132064	0.00266623	0.0137156741
MAPRE2	0.5131603	0.002669	0.0137156741
POLRMT	0.5130611	0.002674964	0.0137156741
DRG1	0.51288515	0.002685573	0.0137266336
LOC285014	0.51277995	0.002691933	0.0137266336
ASMTL	0.5126406	0.002700378	0.0137318672
SV2A	0.5124062	0.002714637	0.0137457637
CCDC131	0.51235163	0.002717963	0.0137457637
ACVR1	0.51213056	0.002731483	0.0137681404
UCP1	0.5120363	0.002737264	0.0137681404
MAGEH1	0.51173717	0.002755686	0.0137814621
RPL34	0.5116218	0.002762821	0.0137814621
EEF1G	0.51154155	0.002767791	0.0137814621
CCNB1IP1	0.5114397	0.002774112	0.0137814621
PLXND1	0.51128334	0.002783838	0.0137814621
SUPT3H	0.51127136	0.002784585	0.0137814621
C1orf181	0.51094776	0.002804814	0.0138131636
RPL10	0.5109098	0.002807196	0.0138131636
ZNF124	0.5108114	0.002813378	0.0138131636
ATP1B3	0.51049256	0.002833487	0.0138591745

ARIH2	0.5104256	0.002837724	0.0138591745
MAGED2	0.5101033	0.002858208	0.0139224816
RNPS1	0.50964993	0.002887233	0.0139705200
CP	0.5092233	0.002914781	0.0139705200
MTG1	0.50920355	0.002916061	0.0139705200
DKC1	0.5091831	0.002917387	0.0139705200
SMARCC1	0.50913894	0.002920254	0.0139705200
USP53	0.5090679	0.002924871	0.0139705200
TRIM28	0.5090676	0.002924891	0.0139705200
AKAP5	0.50892925	0.002933899	0.0139705200
PAICS	0.50889707	0.002935998	0.0139705200
THAP5	0.5086957	0.00294916	0.0139971671
CUEDC2	0.50823367	0.002979557	0.0141052686
NTHL1	0.50787514	0.00300333	0.0141167523
RAP1GDS1	0.50757194	0.003023563	0.0141167523
TARBP1	0.5075587	0.003024448	0.0141167523
ACBD6	0.5075559	0.003024636	0.0141167523
LOC144097	0.5074784	0.003029827	0.0141167523
POMP	0.50742334	0.003033521	0.0141167523
ZFP28	0.5071922	0.003049068	0.0141167523
ST6GALNAC6	0.5069882	0.003062845	0.0141167523
NKRF	0.5068905	0.003069466	0.0141167523
NPIP	0.5068874	0.003069676	0.0141167523
ANKS3	0.5068451	0.003072543	0.0141167523
PEA15	0.506792	0.003076149	0.0141167523
ZNF581	0.50671875	0.003081128	0.0141167523
MAGED4///MAGED4B	0.50649434	0.003096426	0.0141518136
RPL24	0.50587124	0.003139247	0.0142478168
FADS1	0.5058584	0.003140133	0.0142478168
C6orf134	0.50579613	0.003144443	0.0142478168
USP40	0.5057416	0.003148221	0.0142478168
YARS	0.5055299	0.003162923	0.0142619820
SH3PXD2A	0.50547475	0.003166761	0.0142619820
C1orf165	0.5052595	0.003181784	0.0142948597
PDCD2L	0.5049272	0.003205098	0.0143647370
KLHL24	0.5046917	0.00322171	0.0144043121
CAPRIN1	0.50457907	0.003229683	0.0144051644
DDX31	0.50410837	0.00326318	0.0144998838
MAP4K5	0.5039888	0.003271737	0.0144998838
GAGE2C	0.5039514	0.00327442	0.0144998838
RAG1AP1	0.50364816	0.003296221	0.0145422675
ARMCX1	0.50345284	0.003310332	0.0145422675
CYCS	0.5034124	0.003313257	0.0145422675
TBC1D19	0.5033826	0.003315417	0.0145422675
SYNC1///RBBP4	0.50275743	0.003360993	0.0147073240

PLA2G6	0.50264734	0.003369074	0.0147079150
CRIM1	0.5025381	0.003377111	0.0147083117
IGHM	0.5021437	0.00340626	0.0148004396
FLJ40142	0.5020033	0.003416687	0.0148109781
TNFSF4	0.5018451	0.00342847	0.0148273317
UBE3A	0.5011726	0.003478958	0.0150106090
PSMA6	0.5008447	0.003503805	0.0150826583
NOLA2	0.5006693	0.00351716	0.0151050189
RARRES3	0.4998543	0.00357979	0.0152934884
LTBP3	0.49978495	0.003585164	0.0152934884
SPOCK2	0.49976867	0.003586425	0.0152934884
PTGES3	0.49965388	0.003595338	0.0152934884
TOMM22	0.49956366	0.003602356	0.0152934884
LOC100131138	0.49935004	0.00361902	0.0153265581
PHLDB2	0.49905646	0.00364203	0.0153265581
ATXN2L	0.49900842	0.003645807	0.0153265581
MN1	0.49890524	0.003653931	0.0153265581
PRTFDC1	0.49889788	0.003654511	0.0153265581
PPA1	0.49863866	0.003674994	0.0153265581
HS6ST1	0.49861798	0.003676632	0.0153265581
GTPBP6	0.49851218	0.003685023	0.0153265581
PRPSAP2	0.49841672	0.003692608	0.0153265581
MAST4	0.49841246	0.003692947	0.0153265581
PEX1	0.49810854	0.003717189	0.0153265667
NR1D1	0.49805558	0.003721427	0.0153265667
RPS24	0.49796057	0.003729041	0.0153265667
THAP4	0.49784043	0.003738688	0.0153265667
KPNA5	0.4978129	0.003740902	0.0153265667
NAP1L1	0.4977914	0.00374263	0.0153265667
ZNF74	0.4971514	0.003794431	0.0154883399
PFKP	0.4970506	0.003802645	0.0154883399
KLHL25	0.4969576	0.003810239	0.0154883399
ECHS1	0.49689198	0.003815604	0.0154883399
ZNF43	0.4966639	0.003834303	0.0155301857
PSMG1	0.49631688	0.003862904	0.0156118675
DMAP1	0.49616373	0.003875585	0.0156289931
ME3	0.49588314	0.003898911	0.0156888788
KLF9	0.49568883	0.003915134	0.0156905112
SFRS2B	0.49565428	0.003918025	0.0156905112
KLC1	0.49557403	0.003924747	0.0156905112
IMMP2L	0.49543834	0.003936135	0.0156905807
SCN2A	0.49537194	0.003941718	0.0156905807
TOMM20	0.49505067	0.003968827	0.0157287841
PBLD	0.4949702	0.003975642	0.0157287841

LOC100131564///LOC100130531	0.49475753	0.003993703	0.0157287841
ZNF709	0.49471173	0.003997603	0.0157287841
LETMD1	0.49468446	0.003999925	0.0157287841
PHLDA3	0.4946566	0.0040023	0.0157287841
C6orf35	0.49447298	0.004017978	0.0157569434
CEP70	0.49415	0.004045685	0.0158171171
BXDC2	0.49408966	0.00405088	0.0158171171
USP13	0.49381977	0.004074185	0.0158171171
ADFP	0.4938085	0.00407516	0.0158171171
LOC100128545	0.49379826	0.004076048	0.0158171171
HOXA10	0.49364963	0.004088936	0.0158339342
ERCC1	0.49321467	0.004126855	0.0159474084
RCC1	0.49300152	0.004145547	0.0159862656
GNPNAT1	0.49279925	0.004163352	0.0160215479
TP53I13	0.49212348	0.004223316	0.0162185849
PFDN2	0.4919398	0.004239743	0.0162479592
TINP1	0.4917543	0.00425639	0.0162780535
KHDC1	0.49148703	0.004280471	0.0163363955
ZNF608	0.49134994	0.004292868	0.0163499973
ILF2	0.49123976	0.004302854	0.0163543794
PITPNB	0.4911045	0.004315139	0.0163568819
PEBP1	0.49094772	0.004329419	0.0163568819
BCAT1	0.49085987	0.004337439	0.0163568819
SLC27A1	0.49071532	0.00435066	0.0163568819
TTC3	0.49058032	0.004363041	0.0163568819
ABL1	0.49055403	0.004365455	0.0163568819
USP22	0.49052083	0.004368505	0.0163568819
YWHAG	0.49031588	0.004387379	0.0163568819
HSPA9	0.49030593	0.004388297	0.0163568819
WDR77	0.490223	0.004395956	0.0163568819
NPDC1	0.4901715	0.004400717	0.0163568819
PIAS2	0.49002823	0.004413985	0.0163733191
NIP7	0.48987204	0.004428491	0.0163942737
BRF1	0.48966944	0.004447366	0.0164091924
SNRPE	0.48963854	0.004450251	0.0164091924
BTF3	0.4892899	0.004482913	0.0164967633
C5orf42	0.48864773	0.004543615	0.0166759742
DDAH1	0.4885845	0.004549631	0.0166759742
DLL1	0.4884303	0.004564328	0.0166967809
HEMK1	0.48799425	0.004606108	0.0167764841
CIRH1A	0.48789433	0.004615729	0.0167764841
C1orf57	0.48787665	0.004617432	0.0167764841
TRIO	0.48782545	0.00462237	0.0167764841
RPL29	0.4876897	0.004635484	0.0167911563

TIMM9	0.48752746	0.004651199	0.0168151745
TPSG1	0.48697713	0.004704846	0.0169759648
CNNM4	0.4864695	0.004754799	0.0170954708
SYNCRIP	0.486395	0.00476217	0.0170954708
ARD1A	0.48634565	0.004767057	0.0170954708
LOC157627	0.48626643	0.00477491	0.0170954708
LOC286191	0.48610947	0.004790506	0.0171181981
PECI	0.4859685	0.004804548	0.0171352955
EIF5A	0.4856515	0.004836256	0.0171792584
ADNP2	0.48561198	0.004840221	0.0171792584
HRAS	0.48555344	0.004846099	0.0171792584
DNMT3A	0.48547488	0.004853999	0.0171792584
WBP11	0.48537192	0.004864369	0.0171831050
MCCC2	0.48517528	0.004884225	0.0171937771
TTYH1	0.48515806	0.004885968	0.0171937771
PITRM1	0.48493776	0.004908304	0.0172141880
ARHGAP22	0.48488805	0.004913356	0.0172141880
SYNJ2BP	0.484826	0.004919668	0.0172141880
CD99	0.48459357	0.004943376	0.0172438118
ENTPD6	0.4845441	0.004948434	0.0172438118
CST9L	0.48441657	0.004961494	0.0172438118
KIAA1166	0.48437852	0.004965398	0.0172438118
HOXC4	0.48363793	0.005041881	0.0174766325
BIRC3	0.48349124	0.00505715	0.0174967937
JUP	0.483305	0.005076591	0.0175312872
CACNB3	0.48299387	0.005109215	0.0175552692
MCCC1	0.48291302	0.005117722	0.0175552692
TGIF2	0.4828901	0.005120135	0.0175552692
LSM7	0.48285097	0.005124258	0.0175552692
ARHGEF19	0.48271605	0.005138496	0.0175552692
RPL10A	0.48262808	0.005147799	0.0175552692
RPSA	0.482608	0.005149925	0.0175552692
PCK2	0.48212966	0.005200774	0.0176960159
KIF3A	0.48168272	0.005248675	0.0178262338
NET1	0.48144108	0.005274729	0.0178356719
WARS2	0.48143548	0.005275335	0.0178356719
C1orf2	0.481389	0.005280361	0.0178356719
ALDH18A1	0.4807889	0.005345605	0.0180001505
MAGED1	0.48076236	0.005348505	0.0180001505
SLC24A1	0.48064333	0.005361534	0.0180112512
FKBP5	0.48029774	0.005399516	0.0181059857
ATF5	0.479969	0.005435862	0.0181919220
ZNF446	0.47988844	0.0054448	0.0181919220
C9orf85	0.47966176	0.005470017	0.0182432459
ERO1LB	0.47953296	0.005484391	0.0182582873

C1orf105	0.47921854	0.005519614	0.0183425593
NPDC1	0.47889233	0.005556364	0.0184315946
DEAF1	0.47875726	0.00557164	0.0184492051
SCHIP1	0.47843716	0.00560799	0.0184673846
AHNAK	0.47839102	0.005613246	0.0184673846
TMEM39B	0.4783107	0.005622405	0.0184673846
FAM60A	0.4782714	0.005626893	0.0184673846
COQ10A	0.47823757	0.005630757	0.0184673846
C10orf76	0.47808704	0.00564798	0.0184673846
SRRM1	0.47802067	0.005655588	0.0184673846
GALNT5	0.47800884	0.005656946	0.0184673846
SSRP1	0.47750413	0.0057151	0.0186022475
PSD3	0.477476	0.005718357	0.0186022475
ZNF91	0.4773178	0.005736698	0.0186164778
MAP4K1	0.4772005	0.005750331	0.0186164778
LSM2	0.4771784	0.005752904	0.0186164778
RPL4	0.47672236	0.005806189	0.0187561184
PNO1	0.47645727	0.005837357	0.0187906547
SIGIRR	0.4762507	0.005861742	0.0187906547
SLC9A2	0.47622654	0.005864601	0.0187906547
PLCXD1	0.47605935	0.00588441	0.0187906547
CAPRIN1	0.4758673	0.005907235	0.0187906547
EZR	0.47585535	0.005908658	0.0187906547
TTC19	0.4757863	0.005916885	0.0187906547
NDUFS4	0.47575969	0.005920059	0.0187906547
RALY	0.47574133	0.005922248	0.0187906547
ZBED4	0.47572714	0.005923941	0.0187906547
SERTAD4	0.47568855	0.005928548	0.0187906547
PARP6	0.47560266	0.005938812	0.0187910103
LDHB	0.47514915	0.00599326	0.0189292468
MYO1B	0.47506878	0.006002954	0.0189292468
DDX50	0.47462678	0.006056503	0.0190578834
HLA-DMA	0.47456238	0.00606434	0.0190578834
CASKIN2	0.47286323	0.006274247	0.0196402119
PHYH	0.47254753	0.006313922	0.0196402119
CLIC5	0.47249925	0.006320008	0.0196402119
HLA-DRB5	0.4724609	0.006324847	0.0196402119
TSEN54	0.472404	0.006332031	0.0196402119
SH3PXD2B	0.47236663	0.006336753	0.0196402119
RPP40	0.4723342	0.006340853	0.0196402119
TPT1	0.47232854	0.006341569	0.0196402119
DIS3L2	0.47230035	0.006345136	0.0196402119
SH3PXD2A	0.4721946	0.006358529	0.0196488100
GTF2F1	0.4720082	0.006382199	0.0196564373
SIAH1	0.4720079	0.006382236	0.0196564373

STRAP	0.47174338	0.006415953	0.0197249835
LRRC41	0.4716663	0.006425805	0.0197249835
HLA-DRA	0.47141954	0.006457437	0.0197892647
MYH3	0.4711521	0.00649187	0.0198619031
TCEAL1	0.47098982	0.006512838	0.0198657889
PSME1	0.47097617	0.006514605	0.0198657889
PDE6D	0.47067803	0.006553289	0.0199325041
OCIAD2	0.47064167	0.00655802	0.0199325041
ZNF608	0.47026506	0.006607196	0.0200406952
HAPLN1	0.47020346	0.00661527	0.0200406952
CLN8	0.4698403	0.006663038	0.0200934946
EIF3C	0.46971223	0.006679951	0.0200934946
LOC728442	0.46959928	0.0066949	0.0200934946
SNRPD1	0.46959552	0.006695397	0.0200934946
GLS	0.46955943	0.00670018	0.0200934946
ABLIM1	0.46955103	0.006701294	0.0200934946
PRR3	0.4694953	0.006708687	0.0200934946
DDX54	0.46923518	0.006743281	0.0201453495
FABP4	0.46920153	0.006747767	0.0201453495
BOP1	0.46911955	0.006758708	0.0201455209
PPP3CC	0.46886525	0.006792743	0.0202144169
TCF7L2	0.46871918	0.006812356	0.0202241447
SMAD3	0.46867824	0.006817864	0.0202241447
RPS16	0.46836364	0.006860302	0.0202569054
SELS	0.46829543	0.006869535	0.0202569054
JOSD1	0.468272	0.006872707	0.0202569054
NIPSNAP1	0.46809992	0.006896052	0.0202569054
C4orf28	0.46800575	0.006908856	0.0202569054
LOC650794	0.46800056	0.006909562	0.0202569054
FATE1	0.46790144	0.006923062	0.0202569054
GPR116	0.46790078	0.006923151	0.0202569054
PRTFDC1	0.4678696	0.006927402	0.0202569054
SHFM1	0.4677112	0.006949033	0.0202881074
RPS13	0.46739635	0.006992203	0.0203615646
RPL23A	0.46736732	0.006996194	0.0203615646
FADS3	0.46717644	0.00702249	0.0204041184
SDCCAG3	0.4671013	0.007032862	0.0204041184
CIB2	0.4669326	0.007056203	0.0204397993
C1orf142	0.4667986	0.007074787	0.0204616105
PMAIP1	0.46665114	0.007095287	0.0204888865
IPO5	0.46655083	0.007109262	0.0204941772
ZMYND11	0.46640924	0.007129027	0.0204941772
SNHG8	0.46639988	0.007130335	0.0204941772
NAV2	0.46617582	0.007161711	0.0205524451
DNASE2	0.4660892	0.007173875	0.0205554839

GUCY1A2	0.46557495	0.007246438	0.0207313087
MAPKAPK5	0.46502367	0.007324921	0.0209235012
XPO4	0.46487632	0.00734602	0.0209235027
PHB	0.46481892	0.007354253	0.0209235027
GTF3C5	0.46474016	0.007365564	0.0209235027
NDUFV1	0.46465692	0.007377533	0.0209235027
PLEKHO1	0.46462977	0.007381441	0.0209235027
RCN2	0.4644918	0.007401323	0.0209477812
PTK7	0.4643007	0.007428943	0.0209938527
SNRPD2	0.46416777	0.007448203	0.0210161947
FAM122C	0.4640263	0.00746875	0.0210357378
HSPBP1	0.46386415	0.007492359	0.0210357378
MED6	0.46372083	0.007513279	0.0210357378
FBXL10	0.4636564	0.0075227	0.0210357378
FAM62A	0.46346995	0.007550018	0.0210357378
ERGIC3	0.46344262	0.00755403	0.0210357378
GMDS	0.46336627	0.007565247	0.0210357378
MAPRE2	0.46319425	0.00759057	0.0210357378
SLC25A33	0.4631885	0.007591418	0.0210357378
ITPA	0.46297032	0.007623643	0.0210357378
C1orf68	0.46290827	0.007632828	0.0210357378
KLHL3	0.46269137	0.007665012	0.0210357378
LOC642361	0.46261224	0.007676782	0.0210357378
TIMM44	0.46256024	0.007684525	0.0210357378
MYO5C	0.46255925	0.007684672	0.0210357378
NT5C	0.4625103	0.007691969	0.0210357378
DFNB31	0.4624991	0.007693635	0.0210357378
SP6	0.46249035	0.007694942	0.0210357378
SEMA3F	0.46238187	0.007711135	0.0210357378
RPL14	0.46234104	0.007717237	0.0210357378
P2RX4	0.46224418	0.00773173	0.0210357378
MED28	0.4622348	0.007733136	0.0210357378
LRP8	0.46216723	0.00774326	0.0210357378
TMEM67	0.46206105	0.007759195	0.0210357378
PSORS1C2	0.46203387	0.007763278	0.0210357378
ABT1	0.46188635	0.007785472	0.0210357378
MMD	0.46184972	0.007790991	0.0210357378
IDUA	0.46176693	0.007803477	0.0210357378
TMEM19	0.46173573	0.007808188	0.0210357378
PBX4	0.46164408	0.007822037	0.0210357378
XBP1	0.46162546	0.007824854	0.0210357378
PDE8B	0.46160617	0.007827771	0.0210357378
RDH14	0.4615182	0.007841094	0.0210357378
ST13	0.46151537	0.007841523	0.0210357378
CNOT2	0.4613818	0.007861788	0.0210595797

TMCO4	0.46120974	0.007887957	0.0210991451
ERO1LB	0.46107644	0.007908283	0.0211139516
SPATS2	0.4610239	0.007916306	0.0211139516
MRTO4	0.4605919	0.007982538	0.0212599681
NDUFAF2	0.46017718	0.008046566	0.0213800948
DNAJC21	0.46014127	0.00805213	0.0213800948
PI4KAP1	0.46007574	0.008062294	0.0213800948
LCT	0.4599469	0.008082306	0.0213908980
TSPY1	0.459884	0.008092093	0.0213908980
JPH4	0.45982656	0.008101037	0.0213908980
ABL2	0.45963532	0.008130878	0.0214244153
HOMER1	0.45954803	0.008144531	0.0214244153
ZNF553	0.45952296	0.008148454	0.0214244153
HLA-F	0.45931366	0.008181281	0.0214802144
TNFRSF21	0.45923254	0.008194034	0.0214832251
NHP2L1	0.4590171	0.008227986	0.0214970798
ANAPC5	0.458973	0.008234952	0.0214970798
UBXD4	0.45895094	0.008238437	0.0214970798
FLJ20160	0.45885265	0.008253982	0.0214970798
RPL17	0.45875928	0.008268774	0.0214970798
TXLNB	0.45875785	0.008269001	0.0214970798
KCNJ3	0.458647	0.008286588	0.0215125868
RUVBL1	0.45813596	0.008368092	0.0216578730
LOC100132083	0.45813048	0.00836897	0.0216578730
PDE6G	0.4580253	0.00838583	0.0216578730
NNT	0.45796046	0.008396239	0.0216578730
C1orf122	0.45793048	0.008401055	0.0216578730
EIF3I	0.45764795	0.008446557	0.0217448915
PSTK	0.45738947	0.008488367	0.0218196676
POLR3G	0.4573228	0.008499179	0.0218196676
DLSTP	0.45710266	0.008534967	0.0218623828
tcag7.1196	0.4570043	0.008550996	0.0218623828
FAM101A	0.45697582	0.008555644	0.0218623828
EIF3F	0.45688933	0.008569768	0.0218623828
FAM108B1	0.4568581	0.008574873	0.0218623828
TTYH1	0.45671824	0.008597766	0.0218905982
RPL9	0.45657656	0.008621009	0.0219196259
TARBP2	0.4561257	0.008695328	0.0220782608
DDX56	0.45591965	0.008729475	0.0221193943
C20orf30	0.4558838	0.008735428	0.0221193943
PRRT2	0.45568848	0.00876792	0.0221384421
RNASEH1	0.45544758	0.008808133	0.0221384421
RAB4A	0.4554431	0.008808881	0.0221384421
TUSC3	0.45539582	0.008816795	0.0221384421
FLNB	0.45530555	0.008831917	0.0221384421

LOC90784	0.45522225	0.008845891	0.0221384421
RHOF	0.4551967	0.00885018	0.0221384421
TMEM25	0.45516947	0.008854754	0.0221384421
DDHD2	0.45512763	0.008861787	0.0221384421
PCID2	0.45512307	0.008862553	0.0221384421
RFXDC2	0.454835	0.0089111	0.0221761942
EVL	0.45481673	0.008914187	0.0221761942
TRIM64	0.4547567	0.008924332	0.0221761942
TMEM5	0.45464873	0.00894261	0.0221761942
LOC729658	0.45463282	0.008945307	0.0221761942
PMAIP1	0.45460778	0.00894955	0.0221761942
MORF4L2	0.4543137	0.008999525	0.0222658718
IMP5	0.4542534	0.009009799	0.0222658718
PSMB7	0.45374826	0.009096275	0.0223968521
NDUFV2	0.45374814	0.009096296	0.0223968521
MAGED2	0.45367265	0.009109279	0.0223968521
GKAP1	0.45366004	0.009111448	0.0223968521
LOC100129232///ZNF496	0.45359123	0.009123299	0.0223968521
CREM	0.4534114	0.009154331	0.0224432671
FSD1L	0.45286423	0.009249303	0.0226461109
OLA1	0.45275575	0.00926823	0.0226624752
UBAP2L	0.4525525	0.00930378	0.0227193889
COQ4	0.45235336	0.009338724	0.0227558482
C1QBP	0.45232734	0.009343298	0.0227558482
CCDC74B	0.45223942	0.009358767	0.0227635712
C12orf10	0.45179626	0.00943707	0.0229239063
NDUFS8	0.45148608	0.009492206	0.0230000735
TCTA	0.45147836	0.009493582	0.0230000735
ACOT7	0.45141038	0.009505703	0.0230000735
VARS2	0.45130044	0.009525334	0.0230174846
AKR1A1	0.45113748	0.009554496	0.0230186922
WBSCR22	0.45107344	0.009565977	0.0230186922
MAP4	0.4510561	0.009569089	0.0230186922
EXOC5	0.45101994	0.009575577	0.0230186922
TCERG1	0.4506434	0.009643376	0.0231478387
POLR1D	0.45058286	0.009654312	0.0231478387
ENDOG	0.4503881	0.009689571	0.0232023233
RIBC1	0.45022988	0.009718296	0.0232219366
CACNA1A	0.45020482	0.009722853	0.0232219366
LAPTM4A	0.44995028	0.009769234	0.0233026445
KIAA0841	0.4495599	0.00984073	0.0234407622
KLHL5	0.4494856	0.009854387	0.0234407622
CPT1A	0.44942722	0.009865129	0.0234407622
SETD2	0.44929403	0.009889673	0.0234689548
AHNAK2	0.44920248	0.009906576	0.0234789657

OR1F1	0.44907787	0.009929619	0.0235034844
TCF12	0.44883305	0.009975025	0.0235793153
GRAMD1C	0.44876793	0.009987133	0.0235793153
MTA1	0.44862366	0.010013999	0.0236126269
FDX1	0.44839054	0.010057541	0.0236293006
RPL11	0.4483325	0.010068409	0.0236293006
RPP14	0.44831732	0.010071251	0.0236293006
ZNF131	0.4483126	0.010072133	0.0236293006
EIF4EBP1	0.44792536	0.010144906	0.0237077481
LRRC23	0.4479243	0.010145108	0.0237077481
POLR3D	0.44790956	0.010147884	0.0237077481
ATP5SL	0.44786227	0.010156804	0.0237077481
SOHLH2	0.44769242	0.010188893	0.0237126517
HLA-DPA1	0.4476808	0.010191092	0.0237126517
RFXDC2	0.4476478	0.010197337	0.0237126517
RWDD2B	0.4475534	0.010215224	0.0237244412
HLA-DRB4	0.44738838	0.010246551	0.0237614329
LOC730051	0.44733435	0.010256826	0.0237614329
C7orf40	0.44711664	0.010298315	0.0237696983
PDRG1	0.44710672	0.010300209	0.0237696983
TMEM161A	0.4470873	0.010303919	0.0237696983
SUPV3L1	0.44704625	0.01031176	0.0237696983
HNF4G	0.4469068	0.010338438	0.0238015532
VANGL2	0.44668627	0.01038075	0.0238397914
IDS	0.44668597	0.010380806	0.0238397914
SPRR2C	0.44652593	0.010411602	0.0238403927
LOC149832	0.44651073	0.010414531	0.0238403927
SMAD3	0.44648388	0.010419707	0.0238403927
PHGDH	0.4462854	0.010458034	0.0238985444
TMEM159	0.44602147	0.010509181	0.0239734795
LLGL1	0.4459826	0.010516729	0.0239734795
CD274	0.4457438	0.010563218	0.0240466980
SMARCE1	0.44568428	0.010574831	0.0240466980
TNFRSF11A	0.4454895	0.010612914	0.0241036857
CD70	0.44538742	0.010632916	0.0241195190
BCAT1	0.44515735	0.010678119	0.0241909995
KIAA1683	0.4450714	0.010695047	0.0241909995
RUNX1	0.44499695	0.010709728	0.0241909995
CNDP2	0.4449024	0.0107284	0.0241909995
RADIL	0.4447596	0.010756645	0.0241909995
INPP4B	0.44475952	0.010756663	0.0241909995
TREH	0.44470105	0.010768249	0.0241909995
BBS1	0.4446679	0.010774819	0.0241909995
CCL23	0.44463146	0.01078205	0.0241909995
SCN2A	0.4444173	0.010824617	0.0242571018

BMI1	0.4441471	0.010878523	0.0242768625
PROCA1	0.44414502	0.01087894	0.0242768625
ZCCHC18	0.44398257	0.010911462	0.0242768625
GRASP	0.44398072	0.010911832	0.0242768625
IPO7	0.44396466	0.010915052	0.0242768625
SFRS14	0.4438846	0.010931112	0.0242768625
CDK4	0.44384897	0.010938269	0.0242768625
PDCD7	0.44378915	0.010950289	0.0242768625
C17orf79	0.44378325	0.010951475	0.0242768625
CCDC124	0.44366112	0.010976054	0.0243022440
RPUSD2	0.44355446	0.010997557	0.0243207623
DDX10	0.44311747	0.011086029	0.0244428602
RABL2A	0.44302416	0.011104998	0.0244428602
OR6M1	0.44294927	0.011120243	0.0244428602
SETD4	0.44291762	0.011126691	0.0244428602
IL11	0.44284627	0.011141237	0.0244428602
SENP7	0.44277573	0.011155634	0.0244428602
ROM1	0.4427749	0.011155805	0.0244428602
TNNC1	0.44276214	0.01115841	0.0244428602
SH2D2A	0.4425455	0.011202733	0.0245109442
WDR42A	0.4424612	0.011220023	0.0245197905
SAMD10	0.44237655	0.011237402	0.0245288103
EFHC1	0.4421031	0.011293711	0.0246187704
ANKRD28	0.44204736	0.011305216	0.0246187704
ADAT2	0.44187453	0.011340954	0.0246540860
ARPC5L	0.44184017	0.011348072	0.0246540860
ACYP1	0.44174266	0.011368289	0.0246690539
CD72	0.4414729	0.011424379	0.0247389765
RTKN	0.44145915	0.011427242	0.0247389765
ARHGAP21	0.4410063	0.011521941	0.0249070063
ETFB	0.44095948	0.011531769	0.0249070063
CLSTN3	0.44060728	0.011605931	0.0250133517
MCTS1	0.44059733	0.011608033	0.0250133517
EIF2AK4	0.44043246	0.011642891	0.0250244060
PEA15	0.44042525	0.011644417	0.0250244060
PBRM1	0.44029558	0.011671899	0.0250244060
COPZ1	0.44028556	0.011674023	0.0250244060
UBAP2	0.44025382	0.01168076	0.0250244060
SENP7	0.44018206	0.011696002	0.0250280921
PI4KA	0.44002128	0.011730212	0.0250454134
ELAVL1	0.44001684	0.011731158	0.0250454134
FDX1	0.4397494	0.011788254	0.0251383158
RFXDC2	0.43947726	0.011846587	0.0252051541
RPL10A	0.43941417	0.011860146	0.0252051541
SNED1	0.43941277	0.011860448	0.0252051541

SPPL2B	0.43918407	0.011909709	0.0252808158
HS2ST1	0.43911937	0.011923677	0.0252814732
CRBN	0.43886217	0.011979336	0.0253441434
PTDSS2	0.43882427	0.011987559	0.0253441434
INF2	0.43879315	0.011994311	0.0253441434
FKBP3	0.43868563	0.012017669	0.0253638803
TAGLN2	0.4385704	0.012042741	0.0253638803
HNRNPR	0.43856114	0.01204476	0.0253638803
PCDHB14	0.4384241	0.01207464	0.0253979075
PRDX6	0.43830708	0.01210021	0.0254228022
NOVA2	0.4382386	0.012115193	0.0254248119
BCR	0.4381772	0.012128638	0.0254248119
MYST1	0.43778	0.012215929	0.0255788287
C17orf48	0.43757993	0.012260095	0.0256260521
VARS	0.43749833	0.012278148	0.0256260521
ZNF263	0.43732288	0.012317038	0.0256260521
GNA13	0.43732128	0.012317395	0.0256260521
PLA2G10	0.4372907	0.012324184	0.0256260521
VPRBP	0.43725732	0.012331598	0.0256260521
SGCE	0.43724024	0.012335393	0.0256260521
CCNG1	0.4368176	0.012429623	0.0257842953
USP13	0.43677378	0.012439425	0.0257842953
GPN1	0.4365997	0.012478436	0.0258362249
INE2	0.43642893	0.012516806	0.0258867127
C3orf32	0.43635315	0.012533866	0.0258930647
SERBP1	0.4361179	0.012586949	0.0259133413
BAT1	0.4360619	0.012599613	0.0259133413
SOD1	0.43605906	0.012600252	0.0259133413
WT1	0.436014	0.012610451	0.0259133413
NDUFA5	0.4359801	0.012618125	0.0259133413
TP53BP1	0.43593794	0.012627679	0.0259133413
TTC23	0.43578163	0.012663149	0.0259573519
LRRC51	0.4354534	0.012737906	0.0260817080
CDK6	0.43525034	0.012784334	0.0261478478
ZNF302	0.4350133	0.012838717	0.0262300940
NLE1	0.43490636	0.012863312	0.0262513677
PPP1CC	0.4347426	0.012901057	0.0262880344
MPRIP	0.43470523	0.012909683	0.0262880344
CNIH3	0.43451428	0.012953834	0.0263273724
MYCN	0.4344609	0.012966198	0.0263273724
MIOX	0.43439782	0.012980825	0.0263273724
USP53	0.43434018	0.012994201	0.0263273724
ZHX3	0.4343147	0.013000118	0.0263273724
TPPP3	0.43409613	0.013050963	0.0263784600
USP39	0.43399584	0.013074349	0.0263784600

RPL28	0.43396252	0.013082126	0.0263784600
LRRC14	0.4339572	0.013083371	0.0263784600
HADH	0.43390056	0.013096599	0.0263784600
EIF1AX	0.43382686	0.013113831	0.0263844578
TRIP12	0.43359143	0.01316901	0.0264409367
KIF27	0.4335852	0.013170472	0.0264409367
BHMT	0.43320146	0.013260831	0.0265934975
CD79B	0.4330443	0.013297985	0.0266391453
FAM108B1	0.43276945	0.013363181	0.0267163187
C1orf54	0.43272668	0.013373349	0.0267163187
CREB3	0.43268585	0.013383062	0.0267163187
PMS2L3	0.43263888	0.013394243	0.0267163187
POP5	0.43244147	0.013441323	0.0267813658
ROR1	0.43226954	0.013482439	0.0268173039
GPR89B	0.43224493	0.013488336	0.0268173039
SERPING1	0.43178785	0.013598197	0.0270067196
NPR2	0.4316574	0.013629687	0.0270402472
RPL5	0.43156388	0.013652301	0.0270561126
FOXP1	0.43145135	0.013679555	0.0270728308
PAPD1	0.43140832	0.013689989	0.0270728308
TPSAB1	0.43109053	0.013767245	0.0271928062
HOOK2	0.43103805	0.013780039	0.0271928062
HDAC5	0.43079776	0.013838745	0.0272795708
CYB5B	0.43037522	0.013942482	0.0273705002
HOXA2	0.43037164	0.013943363	0.0273705002
NELL1	0.4303544	0.013947614	0.0273705002
FOXN4	0.43025735	0.013971536	0.0273705002
DHX30	0.43024838	0.013973749	0.0273705002
MRPL45	0.43024194	0.013975338	0.0273705002
RPS3A	0.4301891	0.013988381	0.0273705002
ANKS6	0.43008196	0.014014862	0.0273933575
TENC1	0.4296941	0.014111077	0.0275098556
LRRC17	0.4296059	0.01413303	0.0275098556
TAF1B	0.4295992	0.0141347	0.0275098556
C6orf106	0.42959297	0.014136252	0.0275098556
TARS	0.42954272	0.014148775	0.0275098556
KCNIP3	0.4293841	0.014188366	0.0275578861
ZNF138	0.4290289	0.01427737	0.0277016896
SYMPK	0.42881632	0.014330857	0.0277763522
RASSF6	0.428554	0.014397092	0.0278755411
LASS6	0.42844427	0.014424874	0.0279001481
FTSJ1	0.42830098	0.014461221	0.0279195392
RPL41	0.42825627	0.014472576	0.0279195392
USP46	0.42822647	0.01448015	0.0279195392
ZC3H7B	0.4278772	0.014569166	0.0280598882

IFT140	0.42782205	0.014583259	0.0280598882
LOC57228	0.42766628	0.014623139	0.0281074042
TBXA2R	0.42750883	0.014663539	0.0281507657
PIK3C2A	0.4274599	0.014676115	0.0281507657
PIK3C2B	0.42720506	0.01474175	0.0282286018
ARHGEF3	0.42718396	0.014747195	0.0282286018
MTHFD1	0.4266326	0.01489008	0.0284334093
XRCC6	0.42661452	0.014894788	0.0284334093
NAAA	0.42656392	0.014907964	0.0284334093
TASP1	0.42653447	0.014915635	0.0284334093
PAIP1	0.42637902	0.014956187	0.0284674383
EXOSC5	0.42634818	0.014964245	0.0284674383
GRAMD1C	0.42611638	0.015024914	0.0285535070
TTLL5	0.4258404	0.015097411	0.0286331569
GPR98	0.42583907	0.015097764	0.0286331569
LUZP1	0.42559776	0.015161397	0.0287244072
FAM3C	0.42533466	0.015231029	0.0288268248
PRMT7	0.42523962	0.015256248	0.0288321336
ACOT2	0.42520672	0.015264987	0.0288321336
RPS15A	0.42512226	0.015287438	0.0288451047
AAMP	0.42493817	0.015336471	0.0289081546
ZNF512B	0.42482018	0.015367965	0.0289247324
ZNF177	0.42478818	0.015376519	0.0289247324
CCDC104	0.42468318	0.015404603	0.0289285851
C9orf131	0.4246619	0.0154103	0.0289285851
WDR42A	0.42460534	0.015425453	0.0289285851
GPX1	0.4244382	0.015470298	0.0289407589
ADRB1	0.42442548	0.015473717	0.0289407589
C4orf14	0.42440638	0.01547885	0.0289407589
FAIM3	0.42430657	0.015505696	0.0289545716
UBR5	0.42426264	0.015517523	0.0289545716
LOC729088	0.42402494	0.015581654	0.0290414838
PCF11	0.42394736	0.015602632	0.0290414838
CTNND1	0.4238849	0.01561954	0.0290414838
GIT1	0.42385787	0.01562686	0.0290414838
TBC1D4	0.4236292	0.015688915	0.0291235695
RPS7	0.42341575	0.01574702	0.0291235695
RRBP1	0.42338932	0.015754228	0.0291235695
ZNF792	0.42337757	0.015757432	0.0291235695
TMEM135	0.42334875	0.015765296	0.0291235695
EIF3B	0.42320132	0.015805574	0.0291235695
LOC284100	0.4231977	0.015806569	0.0291235695
GTF2IRD1	0.42319283	0.015807899	0.0291235695
SNX5	0.4231755	0.015812635	0.0291235695
SLC39A13	0.422633	0.015961649	0.0293687995

CAND2	0.42240605	0.01602433	0.0294548509
COPS7B	0.42222732	0.016073838	0.0295022616
SLC45A3	0.4221979	0.016082	0.0295022616
TERF2	0.4219442	0.016152525	0.0296023008
SCMH1	0.42182517	0.016185703	0.0296063375
KRT222P	0.42176998	0.016201107	0.0296063375
MAP3K6	0.42176422	0.016202712	0.0296063375
TMTC4	0.42161435	0.016244605	0.0296177183
UBE2G2	0.42150134	0.016276253	0.0296177183
CDKN1A	0.42148352	0.016281249	0.0296177183
SSX4	0.42146653	0.016286012	0.0296177183
COMMD2	0.42145607	0.016288945	0.0296177183
STK35	0.42126974	0.01634127	0.0296650334
MTRF1L	0.4212493	0.01634702	0.0296650334
LRCH1	0.42117608	0.016367627	0.0296733375
TAP2	0.42107785	0.016395306	0.0296863362
KARS	0.421031	0.016408522	0.0296863362
NES	0.4209475	0.016432099	0.0296863362
PALM	0.42086092	0.016456572	0.0296863362
DDR1	0.4208134	0.016470013	0.0296863362
ITGB7	0.42080984	0.016471025	0.0296863362
PTPN4	0.42060563	0.016528914	0.0297616924
MRPL15	0.42049077	0.016561547	0.0297799616
SHMT2	0.42044845	0.016573584	0.0297799616
ADCK2	0.42040017	0.016587326	0.0297799616
LOC646934	0.42030156	0.016615424	0.0297940021
ATP5G2	0.4202213	0.016638322	0.0297940021
ATF3	0.4202034	0.016643435	0.0297940021
UBA2	0.42006543	0.016682863	0.0298357289
HLA-DPB1	0.4199733	0.016709236	0.0298529762
KATNAL2	0.41991913	0.016724763	0.0298529762
USP40	0.41983166	0.016749855	0.0298689611
IL17D	0.4197576	0.016771125	0.0298781062
FGD5	0.41965494	0.016800648	0.0299019225
CD74	0.4195528	0.01683006	0.0299254957
EEF2	0.4194514	0.01685931	0.0299259946
BANP	0.41942215	0.01686775	0.0299259946
KALRN	0.41938373	0.016878843	0.0299259946
PPYR1	0.41914845	0.01694693	0.0299845894
ATP5B	0.4191159	0.016956365	0.0299845894
PNO1	0.4191017	0.016960489	0.0299845894
CEP68	0.41891873	0.017013624	0.0300421200
IDH3B	0.41887793	0.017025491	0.0300421200
LRP4	0.41874665	0.017063726	0.0300703509
SCRN2	0.41871148	0.017073981	0.0300703509

RAP1GDS1	0.41857246	0.01711457	0.0301131835
LOC441052	0.41843164	0.017155765	0.0301570000
STMN1	0.418228	0.017215481	0.0301766205
CCDC106	0.41812372	0.017246127	0.0301766205
LSM2	0.4180932	0.017255105	0.0301766205
IMPAD1	0.41808525	0.017257446	0.0301766205
EFNA1	0.41806275	0.01726407	0.0301766205
CA11	0.41806045	0.017264744	0.0301766205
TTYH3	0.4178258	0.017333938	0.0302689804
LY6E	0.41752362	0.017423376	0.0303713594
KIAA0907	0.4174773	0.017437117	0.0303713594
PRDM8	0.41741863	0.017454542	0.0303713594
C3orf26	0.41740632	0.017458199	0.0303713594
ZC3H8	0.41731837	0.017484346	0.0303882859
FAM80B	0.41712758	0.017541181	0.0304471354
BZW2	0.41709432	0.017551104	0.0304471354
ProSAPiP1	0.4170029	0.017578408	0.0304657856
C3orf54	0.41694814	0.017594773	0.0304657856
KPTN	0.41686612	0.017619314	0.0304687343
NRAS	0.41679078	0.017641883	0.0304687343
LAMA5	0.41677752	0.017645858	0.0304687343
CDK9	0.41666475	0.01767969	0.0304987010
PHLDB1	0.41650805	0.01772679	0.0305514789
GNAS	0.41636333	0.017770384	0.0305874482
MAGEB2	0.4163291	0.01778071	0.0305874482
ZNF681	0.4160764	0.017857077	0.0306902967
HIST1H2BB	0.41600937	0.017877378	0.0306966852
MPZL1	0.4159516	0.017894888	0.0306982740
CTNND2	0.41586548	0.017921025	0.0307146456
RAC1	0.41577694	0.017947927	0.0307240510
DNAJC10	0.4157382	0.01795971	0.0307240510
WDR13	0.41551238	0.018028509	0.0308105678
C11orf58	0.41546303	0.018043574	0.0308105678
MRO	0.41534	0.018081175	0.0308315428
RHBDF1	0.41531387	0.018089171	0.0308315428
SAGE1	0.41498196	0.018190978	0.0308828061
PNMA1	0.41497737	0.018192388	0.0308828061
COL11A1	0.41497052	0.018194497	0.0308828061
ADAM30	0.41492763	0.018207688	0.0308828061
KIAA1147	0.41492012	0.018209998	0.0308828061
ASPRV1	0.41488972	0.018219354	0.0308828061
NDRG2	0.41468453	0.01828261	0.0309616753
FOXP1	0.41460717	0.018306509	0.0309738100
CAMSAP1	0.41448137	0.018345421	0.0309770497
QRSL1	0.4144752	0.018347332	0.0309770497

FKBP11	0.41440356	0.018369528	0.0309770497
TPSAB1	0.41438472	0.018375365	0.0309770497
DGKE	0.41428527	0.01840622	0.0310008310
ZDHHC8	0.4142257	0.018424723	0.0310037839
TWSG1	0.41397026	0.018504234	0.0310967278
GLUD1	0.41394037	0.018513557	0.0310967278
BMS1	0.41361645	0.018614838	0.0312384997
FARP2	0.4134306	0.018673154	0.0313079783
ARL3	0.41336071	0.018695122	0.0313164442
ADNP2	0.41319007	0.018748855	0.0313264305
MRPL37	0.41314816	0.018762069	0.0313264305
PDCD5	0.4130817	0.018783042	0.0313264305
UST	0.41307092	0.018786449	0.0313264305
ZNF208	0.41301388	0.018804468	0.0313264305
DIO2	0.41299018	0.018811956	0.0313264305
VPS13C	0.41296616	0.018819552	0.0313264305
RPS8	0.41284913	0.018856592	0.0313302202
SULT1E1	0.41284376	0.018858291	0.0313302202
LRRC8B	0.41279858	0.018872607	0.0313302202
FGFR1OP2	0.41266483	0.018915044	0.0313491088
DNMT3B	0.41264883	0.018920125	0.0313491088
HDAC8	0.41260266	0.018934794	0.0313491088
SFRS11	0.41241693	0.018993903	0.0314188690
RLN1	0.41221863	0.019057183	0.0314834245
PGAM5	0.41218808	0.019066947	0.0314834245
ADCY6	0.41188425	0.019164281	0.0315698954
TCF7L2	0.4118557	0.019173449	0.0315698954
PIAS3	0.41180152	0.019190855	0.0315698954
SCFD2	0.41177198	0.01920035	0.0315698954
RPL35	0.4116153	0.01925078	0.0315698954
C7orf11	0.41161135	0.019252056	0.0315698954
UPF3A	0.4115537	0.019270638	0.0315698954
RPL35A	0.4115104	0.019284608	0.0315698954
INOC1	0.41150683	0.019285763	0.0315698954
IHPK2	0.41147256	0.019296825	0.0315698954
ENSA	0.41141504	0.019315403	0.0315698954
GUCY1A3	0.41132063	0.019345932	0.0315698954
TMEM47	0.41119638	0.019386167	0.0315698954
DRAP1	0.41115987	0.019398002	0.0315698954
USP34	0.4111431	0.019403443	0.0315698954
FGF18	0.41110253	0.019416602	0.0315698954
MRPL48	0.4110938	0.019419437	0.0315698954
BAD	0.4110726	0.019426316	0.0315698954
PSMG2	0.41086406	0.019494124	0.0316363398
TNFSF9	0.41083935	0.01950217	0.0316363398

TAF1B	0.41075325	0.019530231	0.0316363398
GPATCH4	0.4107369	0.019535568	0.0316363398
EIF3J	0.41060475	0.019578708	0.0316784865
RPS15	0.41052526	0.019604696	0.0316928317
CNOT2	0.41026253	0.019690802	0.0317930471
TFPT	0.41023135	0.01970104	0.0317930471
PELI3	0.4100344	0.019765824	0.0318698086
LYRM2	0.40994096	0.019796617	0.0318916780
GNAS	0.4098508	0.019826366	0.0318974628
ZNF639	0.40982565	0.019834673	0.0318974628
GJC2	0.40970075	0.019875964	0.0319240946
ANKRD16	0.4096648	0.019887859	0.0319240946
KIAA0114	0.4095763	0.019917179	0.0319240946
PMVK	0.40953994	0.019929234	0.0319240946
ZMYM3	0.40947	0.01995244	0.0319240946
EIF2AK1	0.40946314	0.019954715	0.0319240946
SELI	0.40940416	0.019974303	0.0319278367
IER5	0.40927005	0.0200189	0.0319599846
DYRK2	0.409213	0.020037893	0.0319599846
VGLL4	0.40918803	0.020046214	0.0319599846
HLA-DRB1	0.40910023	0.020075487	0.0319791105
SSX2	0.40856275	0.020255473	0.0322144383
FAM125A	0.40855515	0.020258026	0.0322144383
ARG2	0.40840814	0.020307492	0.0322653800
WDR4	0.4080554	0.02042659	0.0324019945
SOX5	0.4080498	0.020428486	0.0324019945
HIST1H2AE	0.40764108	0.020567212	0.0325941005
GTPBP8	0.4073919	0.020652171	0.0327007430
POLR3E	0.40725124	0.02070026	0.0327335471
BTBD1	0.40722787	0.020708257	0.0327335471
NUDCD1	0.40698797	0.020790514	0.0328355302
C18orf19	0.40686792	0.020831773	0.0328726444
ZNF253	0.4067896	0.02085873	0.0328804520
RBBP6	0.40675035	0.020872248	0.0328804520
SNRPA	0.40658444	0.020929476	0.0329254276
DDO	0.40656447	0.020936374	0.0329254276
SOX12	0.40650693	0.02095626	0.0329287243
ASCC1	0.40636396	0.021005727	0.0329709336
RABGAP1	0.40629587	0.021029325	0.0329709336
CYTL1	0.406275	0.02103656	0.0329709336
SEPTIN15	0.40615603	0.02107785	0.0330076991
SNPH	0.40604866	0.021115175	0.0330381986
CD109	0.40533826	0.02136349	0.0333290858
SCYE1	0.40531695	0.021370973	0.0333290858
SERPINE1	0.4052545	0.02139292	0.0333290858

SCAMP3	0.40525022	0.021394428	0.0333290858
PCDHB7	0.40522164	0.02140448	0.0333290858
LIPA	0.40518257	0.021418229	0.0333290858
TCP1	0.4051573	0.021427127	0.0333290858
CD2AP	0.40487987	0.021524992	0.0334531992
CATSPER2P1	0.40469548	0.02159024	0.0335264549
PMPCA	0.4046352	0.02161161	0.0335315089
NOL5A	0.40455616	0.02163965	0.0335317518
PEPD	0.40450543	0.021657662	0.0335317518
NAAA	0.40448165	0.021666113	0.0335317518
UCK1	0.404365	0.021707594	0.0335678835
NR1D2	0.4042689	0.021741822	0.0335927483
NONO	0.4041997	0.021766495	0.0336028209
TRAF3IP2	0.4039838	0.021843612	0.0336937715
PSMB1	0.4039286	0.021863366	0.0336961619
C16orf58	0.40380892	0.021906247	0.0337341624
PRKCE	0.40372592	0.021936027	0.0337345210
ATF7IP2	0.40369472	0.021947231	0.0337345210
RAB40B	0.40357655	0.021989703	0.0337345210
MAPKBP1	0.4035272	0.022007462	0.0337345210
RABL2B	0.403506	0.02201509	0.0337345210
SCARF1	0.40350395	0.02201583	0.0337345210
CATR1	0.40345007	0.022035237	0.0337363306
TRIM45	0.40319636	0.022126812	0.0338071985
MECR	0.40317547	0.022134367	0.0338071985
CRBN	0.40317008	0.022136318	0.0338071985
C12orf30	0.40290663	0.022231774	0.0339249907
SLC39A8	0.4028045	0.02226887	0.0339536066
RCBTB1	0.40266752	0.022318704	0.0340015812
CTTNBP2NL	0.4022383	0.022475451	0.0342122202
DTX1	0.40212974	0.022515245	0.0342446331
P4HA2	0.40205386	0.02254309	0.0342588338
TRIM13	0.40195817	0.022578247	0.0342774335
TSSC1	0.40190697	0.022597076	0.0342774335
PLLP	0.40186945	0.022610884	0.0342774335
NUDC	0.40180552	0.022634422	0.0342809544
NPAL3	0.40176257	0.022650247	0.0342809544
CMTM8	0.40166205	0.022687325	0.0343090184
AFG3L2	0.40158165	0.02271702	0.0343258808
WDR27	0.4014261	0.02277455	0.0343847407
DDX31	0.40128353	0.02282739	0.0344122530
POU3F1	0.40127662	0.022829955	0.0344122530
FAM107A	0.4011226	0.022887157	0.0344704049
CMPK1	0.4008901	0.022973724	0.0345726529
MTG1	0.40074056	0.02302955	0.0346144647

SYTL2	0.40071553	0.023038909	0.0346144647
AIRE	0.40048638	0.023124697	0.0347147100
ICAM2	0.4004372	0.02314314	0.0347147100
C9orf102	0.39992574	0.023335697	0.0349752025
LOC285014	0.39972922	0.023410032	0.0350582275
RPL27A	0.3994461	0.023517469	0.0351906509
PES1	0.39923075	0.023599459	0.0352848131
EBF2	0.39908457	0.023655249	0.0353311401
PPAT	0.39904958	0.023668619	0.0353311401
OTUD4	0.39896503	0.02370095	0.0353508932
CRABP2	0.39885545	0.02374291	0.0353538426
SLC25A2	0.39885268	0.023743972	0.0353538426
APCS	0.39881027	0.023760227	0.0353538426
DSEL	0.39867622	0.02381167	0.0354019287
ZNF331	0.3985389	0.023864467	0.0354058172
TAF4B	0.3984352	0.02390439	0.0354058172
SYNPO	0.39842087	0.023909915	0.0354058172
ZNF85	0.39842	0.023910249	0.0354058172
KIAA1529	0.39836857	0.02393008	0.0354058172
COL20A1	0.39830878	0.023953145	0.0354058172
IQCK	0.39829004	0.023960382	0.0354058172
MRPL19	0.3982721	0.023967309	0.0354058172
PPP1R16B	0.3979059	0.02410907	0.0355384823
C18orf19	0.39789882	0.02411181	0.0355384823
TUBA3C	0.39789134	0.024114713	0.0355384823
CHCHD3	0.3976415	0.024211837	0.0356328285
SCFD2	0.39762765	0.024217233	0.0356328285
ELAVL4	0.39746264	0.024281573	0.0356649564
MALL	0.39743724	0.024291484	0.0356649564
ROBO2	0.39742345	0.024296872	0.0356649564
DUSP4	0.39726543	0.024358643	0.0356868600
COL19A1	0.3972647	0.024358936	0.0356868600
MTHFD1L	0.39722607	0.024374055	0.0356868600
GPX1	0.39718813	0.024388913	0.0356868600
S100A13	0.39701045	0.024458598	0.0357236883
GATA4	0.39698848	0.024467224	0.0357236883
MKI67IP	0.39689064	0.024505679	0.0357236883
C9orf52	0.39687294	0.024512641	0.0357236883
ALDH7A1	0.3968608	0.024517413	0.0357236883
LOC648987	0.39682913	0.02452988	0.0357236883
ANKRD23	0.39665553	0.024598286	0.0357527230
UNC84A	0.39651132	0.024655232	0.0357527230
BDH2	0.3964849	0.02466567	0.0357527230
NDUFA12	0.39647734	0.024668664	0.0357527230
TSR1	0.39645243	0.024678517	0.0357527230

ASAH3L	0.39643174	0.0246867	0.0357527230
MRPL24	0.39638746	0.024704227	0.0357527230
PRKRA	0.39637345	0.024709774	0.0357527230
EFCAB7	0.3963384	0.024723655	0.0357527230
CLPP	0.39617342	0.024789086	0.0358193585
AARSD1	0.39610097	0.024817862	0.0358322639
PFN2	0.3960535	0.024836734	0.0358322639
CHRNA4	0.39584994	0.024917781	0.0359211936
DCTN5	0.3956949	0.024979657	0.0359823697
MTHFD2L	0.3956337	0.025004122	0.0359896033
PADI1	0.39532277	0.025128698	0.0361171980
ZNF195	0.39530405	0.025136214	0.0361171980
TWISTNB	0.39521402	0.025172388	0.0361171980
AFAP1L2	0.39517236	0.025189145	0.0361171980
C14orf153	0.3951694	0.025190331	0.0361171980
DUSP10	0.3949009	0.025298541	0.0361965150
JMJD2D	0.39490044	0.025298722	0.0361965150
ANAPC1	0.39486268	0.02531397	0.0361965150
TESK1	0.3947921	0.025342487	0.0361965150
LOC441150	0.3947898	0.025343427	0.0361965150
GNL3	0.39463612	0.025405614	0.0362573566
CLEC2B	0.39446256	0.025476007	0.0363108575
SLMO2	0.39444697	0.025482336	0.0363108575
PSD3	0.39428374	0.025548695	0.0363774111
ETV6	0.39417705	0.025592146	0.0363841147
ALAS2	0.39417565	0.025592716	0.0363841147
ZBTB10	0.39411435	0.025617711	0.0363898912
NAPB	0.39402997	0.025652142	0.0363898912
FDXR	0.39402112	0.025655758	0.0363898912
ADAMTS10	0.3939596	0.025680887	0.0363926385
PSCD2	0.39392015	0.025697017	0.0363926385
FUBP3	0.3938563	0.025723144	0.0364017886
SYNCRIP	0.39366296	0.025802365	0.0364860028
FIGN	0.3934082	0.025907058	0.0366060797
TAF9B	0.39332965	0.025939414	0.0366238408
TATDN1	0.3932663	0.025965534	0.0366327770
ELAC2	0.39312243	0.026024919	0.0366458153
GNAI1	0.39309505	0.026036236	0.0366458153
NDNL2	0.3930628	0.026049567	0.0366458153
LAS1L	0.39305216	0.026053967	0.0366458153
ZBTB5	0.39289582	0.026118692	0.0366847992
PDLIM1	0.3928762	0.026126819	0.0366847992
RPS14	0.39284167	0.02614114	0.0366847992
HSPB3	0.39271796	0.026192484	0.0367290060
GSTM4	0.3925304	0.026270479	0.0368012054

MPV17	0.39249858	0.026283735	0.0368012054
UQCRC2	0.39231512	0.02636025	0.0368804405
SPARCL1	0.39221966	0.02640013	0.0369083388
DKFZP761N09121	0.39209956	0.02645038	0.0369406220
CHD1L	0.3920691	0.026463136	0.0369406220
COX5A	0.39190957	0.026530031	0.0369973420
POLR3G	0.3918769	0.026543744	0.0369973420
LMO7	0.39178085	0.026584106	0.0370257187
ZNF202	0.3916523	0.026638206	0.0370630027
MAP7D3	0.3916221	0.026650922	0.0370630027
PIGG	0.39154327	0.026684152	0.0370811552
EID1	0.39149612	0.026704041	0.0370811552
PNPT1	0.39129108	0.026790684	0.0371735803
TMEM14A	0.39109567	0.026873473	0.0372605232
HOXA5	0.39089683	0.02695793	0.0373496470
STK32C	0.3908311	0.02698589	0.0373604206
NR1D2	0.39059493	0.027086575	0.0374717865
XRRA1	0.39034832	0.027192034	0.0375661514
MEX3C	0.3903405	0.027195377	0.0375661514
CRTC3	0.3902864	0.02721857	0.0375701514
C3orf17	0.3902381	0.02723927	0.0375707070
PRKCE	0.39002812	0.02732946	0.0376670368
KPNA3	0.3898703	0.02739741	0.0377325937
SELI	0.3898017	0.027426975	0.0377452273
COMMD7	0.38969037	0.027475035	0.0377832762
PCDHB5	0.3895497	0.027535852	0.0378387989
AGXT2	0.38947323	0.02756896	0.0378389351
ENAH	0.38945505	0.027576836	0.0378389351
TACC2	0.38926244	0.027660392	0.0379254708
TMEM189	0.3892114	0.027682573	0.0379277888
MMAB	0.38898897	0.027779378	0.0379852363
MPI	0.38898614	0.027780611	0.0379852363
TXN2	0.38897362	0.027786067	0.0379852363
TRAF5	0.38872823	0.027893215	0.0381035727
MDH1	0.38865763	0.027924106	0.0381176403
SPHAR	0.38857988	0.027958157	0.0381359975
ZNF10	0.38845164	0.028014392	0.0381522258
EIF2B1	0.3883944	0.028039528	0.0381522258
B3GNT1	0.38839203	0.028040562	0.0381522258
UBAP2	0.38836485	0.028052501	0.0381522258
CDH11	0.38812605	0.028157584	0.0382670250
C16orf45	0.38802534	0.028201994	0.0382992596
CD9	0.38797387	0.028224714	0.0383020129
HSPB1	0.38788	0.028266191	0.0383115507
MC3R	0.3877266	0.028334074	0.0383115507

TF	0.3877092	0.028341783	0.0383115507
CD40	0.3877063	0.028343065	0.0383115507
LOC51149	0.38767353	0.028357593	0.0383115507
CCDC88A	0.38761723	0.028382557	0.0383115507
KHK	0.387525	0.028423497	0.0383115507
TCERG1	0.3875139	0.02842842	0.0383115507
CCDC6	0.38750592	0.028431969	0.0383115507
MAPK8	0.38749072	0.02843872	0.0383115507
HEYL	0.38724712	0.028547123	0.0384018697
LIMK1	0.38724682	0.028547257	0.0384018697
TRIM22	0.3871941	0.028570762	0.0384055777
PSMC3	0.387068	0.028627044	0.0384533080
P2RX5	0.38701725	0.028649721	0.0384558619
BAD	0.38694447	0.02868227	0.0384716535
H2AFY	0.38630077	0.02897145	0.0388313932
ETV6	0.3861429	0.029042732	0.0388692785
MAOA	0.3860606	0.029079957	0.0388692785
LYPLA3	0.38603327	0.029092325	0.0388692785
LPXN	0.38603106	0.029093323	0.0388692785
PDZD2	0.3860059	0.029104711	0.0388692785
TEAD4	0.38585126	0.029174801	0.0389347921
RPS27L	0.38577202	0.029210769	0.0389547071
IFT81	0.3853435	0.029405907	0.0391268473
SEPW1	0.38533965	0.029407663	0.0391268473
CSPP1	0.3853343	0.029410098	0.0391268473
CLIC6	0.38527706	0.029436251	0.0391268473
EXOSC2	0.38525674	0.029445542	0.0391268473
CBFB	0.38519645	0.029473111	0.0391353863
CBR3	0.38493738	0.02959182	0.0391821264
RABL5	0.3849191	0.029600205	0.0391821264
C19orf66	0.38489458	0.029611466	0.0391821264
CALCRL	0.3848664	0.029624416	0.0391821264
PSMD8	0.38479343	0.029657945	0.0391821264
LOX	0.3847808	0.029663756	0.0391821264
MARCH5	0.38477555	0.029666169	0.0391821264
KPNA4	0.38475057	0.029677656	0.0391821264
CADM1	0.38454127	0.029774072	0.0392814022
MEF2C	0.38449204	0.029796788	0.0392833722
LOC283508	0.3843294	0.029871928	0.0393010556
ICOSLG	0.3843278	0.029872673	0.0393010556
CUL3	0.38432515	0.029873898	0.0393010556
SART1	0.3842728	0.029898113	0.0393049767
ASS1	0.38417342	0.029944142	0.0393375492
RPS21	0.38409996	0.0299782	0.0393543604
ACAT2	0.38390398	0.030069217	0.0394231276

C6orf129	0.38389546	0.03007318	0.0394231276
ZNF273	0.3838367	0.030100523	0.0394310461
TMEM126B	0.3836587	0.030183444	0.0395117078
SYN2	0.38352722	0.030244824	0.0395117617
TRPC5	0.38350916	0.030253263	0.0395117617
C12orf24	0.38348496	0.030264573	0.0395117617
VAMP4	0.38346982	0.030271651	0.0395117617
CRYBB2	0.38343012	0.030290216	0.0395117617
CAMK1D	0.38333273	0.030335803	0.0395208404
HPS4	0.38332403	0.030339878	0.0395208404
ZFHX3	0.38318098	0.030406943	0.0395580317
EIF3M	0.38313583	0.030428136	0.0395580317
PCDHA4	0.38312644	0.030432543	0.0395580317
IHPK2	0.3830134	0.03048566	0.0395961351
REXO4	0.38297305	0.03050464	0.0395961351
COPS6	0.38276803	0.030601216	0.0396619789
KHDC1	0.38271153	0.030627878	0.0396619789
UGT2B15	0.38269514	0.030635616	0.0396619789
DKFZP564O0823	0.38268358	0.030641075	0.0396619789
HCFC1	0.38248646	0.030734263	0.0396629728
DCDC2	0.3824754	0.030739497	0.0396629728
RGS5	0.3824711	0.03074153	0.0396629728
DGKQ	0.38244334	0.030754682	0.0396629728
CNOT7	0.38243178	0.03076016	0.0396629728
RPS21	0.38241014	0.03077041	0.0396629728
PFDN6	0.3822687	0.030837486	0.0397217722
GNAS	0.38214725	0.030895172	0.0397684029
WDR33	0.38204476	0.030943925	0.0397740786
LTBP3	0.38202167	0.03095492	0.0397740786
FLRT1	0.3820025	0.030964045	0.0397740786
SEMA3A	0.3819529	0.030987669	0.0397768206
CDC16	0.3818406	0.031041233	0.0397870324
CDH22	0.38181257	0.031054603	0.0397870324
PARN	0.38180104	0.031060109	0.0397870324
C10orf72	0.38163498	0.031139474	0.0398611109
LOC642929	0.3815425	0.031183744	0.0398901936
C20orf46	0.38122553	0.03133587	0.0400571101
WDR18	0.38113758	0.031378187	0.0400835225
HOXD3	0.3807917	0.03154506	0.0402564053
PRKAB2	0.38076696	0.03155702	0.0402564053
BMPR1B	0.38070002	0.031589407	0.0402699672
LOC116236	0.3805562	0.03165909	0.0403264881
RPS29	0.38051862	0.031677317	0.0403264881
PHF10	0.38039008	0.031739727	0.0403569917
ANKRD13C	0.38036448	0.03175217	0.0403569917

YES1	0.38030538	0.031780902	0.0403569917
ZBED3	0.3802726	0.031796854	0.0403569917
ZNF605	0.3802216	0.031821676	0.0403569917
FAM153B	0.3802002	0.031832095	0.0403569917
LOC51035	0.38005874	0.031901065	0.0404048485
PAICS	0.38003325	0.0319135	0.0404048485
MARCH5	0.37974027	0.03205676	0.0405584845
RPL23	0.37958407	0.03213335	0.0406149490
HIST1H2BN	0.3795598	0.032145273	0.0406149490
PBX3	0.379299	0.03227351	0.0407177281
ODF2	0.37927863	0.032283552	0.0407177281
CTBP1	0.37926024	0.032292612	0.0407177281
PSMB2	0.37917107	0.032336578	0.0407209461
RABEPK	0.37916583	0.032339163	0.0407209461
ITFG2	0.37909093	0.032376133	0.0407267529
ATP5A1	0.37897584	0.03243301	0.0407267529
SMA5	0.37897354	0.032434143	0.0407267529
NUP153	0.3789669	0.032437433	0.0407267529
TRUB2	0.37892196	0.032459665	0.0407267529
ATP8B1	0.37888938	0.03247579	0.0407267529
KLHDC9	0.37880865	0.032515775	0.0407492888
RPS28	0.37875596	0.032541897	0.0407544326
HDAC7	0.37851506	0.032661527	0.0408308103
PRKAG2	0.37850845	0.03266482	0.0408308103
CTDSPL2	0.37849993	0.03266906	0.0408308103
FARSB	0.37796083	0.03293813	0.0411161644
ZFR	0.3779535	0.0329418	0.0411161644
MAD1L1	0.37789997	0.032968618	0.0411219083
SNX5	0.37774572	0.033045996	0.0411906657
NDUFA10	0.37754565	0.033146575	0.0412882304
PCNT	0.37745675	0.03319135	0.0413161996
RBM27	0.3773114	0.033264652	0.0413503586
SH3RF1	0.3772939	0.033273485	0.0413503586
LPP	0.3772695	0.03328581	0.0413503586
BPHL	0.377202	0.03331992	0.0413649711
IL27RA	0.37699097	0.03342673	0.0414548461
TMED1	0.37693366	0.033455785	0.0414548461
C10orf25	0.37692633	0.033459503	0.0414548461
PCCB	0.37674484	0.033551663	0.0414979214
DARS	0.37673506	0.033556633	0.0414979214
KIF9	0.37672544	0.033561528	0.0414979214
STARD4	0.37651917	0.033666536	0.0415999721
ASNA1	0.37640297	0.03372581	0.0416454131
UBE2Q2	0.3761645	0.033847727	0.0417680951
ABCC1	0.37606812	0.0338971	0.0418011540

NIPA1	0.37587675	0.033995304	0.0418943460
CCDC62	0.37581763	0.03402569	0.0419038937
ARHGAP5	0.37563786	0.034118224	0.0419055007
LOC158301	0.37562943	0.03412257	0.0419055007
SMN2	0.37556794	0.034154266	0.0419055007
PNMT	0.37553057	0.034173544	0.0419055007
MSH6	0.37551972	0.034179144	0.0419055007
CHRAC1	0.37551066	0.03418382	0.0419055007
NICN1	0.37548605	0.034196522	0.0419055007
PABPC4	0.3754636	0.03420811	0.0419055007
PLEKHA9	0.37537155	0.034255676	0.0419360161
C4orf16	0.37521252	0.034337968	0.0419910965
FRAP1	0.37519693	0.03434604	0.0419910965
CYP2A13	0.3751012	0.034395665	0.0420240105
RELB	0.37497506	0.034461148	0.0420598984
HN1L	0.37495708	0.034470484	0.0420598984
CTAGE5	0.37483573	0.034533586	0.0421091355
ZNF571	0.37479192	0.03455639	0.0421092020
ZNF136	0.37451273	0.034701996	0.0422588122
FOXJ3	0.37445846	0.03473036	0.0422655466
MAF	0.3743439	0.03479029	0.0423106615
ZBTB10	0.37428862	0.034819238	0.0423180627
TSC22D1	0.37420243	0.034864414	0.0423332264
C8orf33	0.3741678	0.03488258	0.0423332264
ABCB8	0.37413397	0.034900326	0.0423332264
PSMD11	0.37402615	0.034956954	0.0423741466
CNR2	0.37391776	0.035013955	0.0424154651
ADAM3A	0.3738664	0.035040986	0.0424204481
KLF10	0.37358612	0.035188828	0.0425592330
MLH3	0.37356192	0.035201613	0.0425592330
SLMO1	0.37337944	0.03529816	0.0426481032
RASL10A	0.37332618	0.03532638	0.0426543571
DPH1	0.37319908	0.035393804	0.0426691653
AHR	0.37318337	0.03540214	0.0426691653
UBE2N	0.37317055	0.035408944	0.0426691653
MEX3A	0.3731293	0.035430852	0.0426691653
RBBP7	0.37307575	0.035459317	0.0426756800
PDE1B	0.37292436	0.035539877	0.0427448423
DEAF1	0.37276146	0.035626728	0.0427967224
METTL7A	0.37275672	0.035629254	0.0427967224
PELO	0.37266618	0.035677604	0.0428159093
NENF	0.3726402	0.03569149	0.0428159093
ZBTB25	0.37257132	0.035728313	0.0428323234
DLST	0.3722392	0.035906322	0.0430147461
CAPN11	0.3722008	0.035926957	0.0430147461

DSP	0.3720609	0.036002185	0.0430769518
MAST4	0.37196007	0.03605648	0.0430946414
CCDC138	0.371947	0.036063533	0.0430946414
GNPTAB	0.37182873	0.036127314	0.0431028315
HCG8///C6orf12	0.37174627	0.036171842	0.0431028315
TMEM159	0.37174276	0.036173742	0.0431028315
GABPB2	0.37173852	0.03617603	0.0431028315
EXOSC7	0.37171856	0.036186818	0.0431028315
TRIM31	0.37166384	0.0362164	0.0431103257
ITGB1	0.37154892	0.03627859	0.0431307846
GSTA1	0.37154597	0.03628019	0.0431307846
HISPPD2A	0.37145382	0.036330123	0.0431624247
USP29	0.3713342	0.03639503	0.0431952143
SLC35E1	0.3712281	0.03645267	0.0431952143
DIRAS1	0.37122652	0.03645353	0.0431952143
CSNK2A2	0.371221	0.036456525	0.0431952143
OBFC1	0.37118813	0.036474403	0.0431952143
FAM80B	0.37107423	0.0365364	0.0431963000
ARL2BP	0.3710625	0.036542796	0.0431963000
KIF7	0.37105784	0.03654533	0.0431963000
SHOX	0.37099114	0.036581676	0.0431973568
CTSF	0.37095824	0.03659962	0.0431973568
ENOPH1	0.37092778	0.036616236	0.0431973568
FHIT	0.37074357	0.036716856	0.0432716348
MGC29506	0.3707141	0.036732975	0.0432716348
TMEM67	0.3706842	0.03674933	0.0432716348
METTL5	0.37050292	0.036848642	0.0433086663
STOX1	0.37045896	0.03687276	0.0433086663
POLR2I	0.37045607	0.036874343	0.0433086663
TAF11	0.37041545	0.03689664	0.0433086663
GOSR2	0.3704134	0.036897767	0.0433086663
SLC5A2	0.3703295	0.03694385	0.0433352765
ABCC4	0.37025073	0.03698716	0.0433540189
CD109	0.37021527	0.037006672	0.0433540189
WDR6	0.37013903	0.037048645	0.0433757381
CLIP2	0.36997148	0.037141025	0.0434440574
PRKCQ	0.3699481	0.03715394	0.0434440574
ATP5J	0.36990523	0.037177607	0.0434442870
YY1	0.36984852	0.037208945	0.0434534746
MYL5	0.3696587	0.037313983	0.0435486649
HLA-DRB6	0.36935377	0.03748323	0.0437006714
OLFML2A	0.36930948	0.037507858	0.0437006714
CHST10	0.36929655	0.037515055	0.0437006714
ALG5	0.3691979	0.037569977	0.0437371242
MTA3	0.3690446	0.037655458	0.0437979272

ADCY1	0.3690194	0.03766953	0.0437979272
ANKS6	0.36887345	0.037751075	0.0438003333
C14orf79	0.36883676	0.037771598	0.0438003333
HSF2	0.368819	0.037781537	0.0438003333
C10orf18	0.3687908	0.037797313	0.0438003333
C16orf86	0.3687773	0.03780487	0.0438003333
SLC6A3	0.36875525	0.03781722	0.0438003333
PSMA5	0.36871925	0.037837386	0.0438003333
ASCC3	0.3686773	0.037860904	0.0438003333
GALP	0.36847776	0.037972882	0.0438939774
SLC5A6	0.3684486	0.037989277	0.0438939774
DDO	0.36837843	0.03802873	0.0439121517
CLIC5	0.3681374	0.038164522	0.0440414777
FAM82A2	0.36809272	0.038189735	0.0440431149
NRAS	0.36804172	0.038218528	0.0440488763
DIRAS3	0.36793825	0.038277008	0.0440888250
DCTN6	0.3677227	0.038399067	0.0441527803
LETM1	0.36770514	0.03840902	0.0441527803
PDGFRL	0.36768952	0.038417876	0.0441527803
LINGO2	0.3676436	0.03844393	0.0441527803
HDAC2	0.36762974	0.0384518	0.0441527803
NARS2	0.3675604	0.03849118	0.0441705978
ITGB1	0.36750194	0.03852439	0.0441813172
APOL3	0.36743706	0.038561285	0.0441962468
HOMER1	0.36715144	0.038724054	0.0443553366
PRKCE	0.3671066	0.03874966	0.0443572175
ATF2	0.36706382	0.038774095	0.0443577564
JTV1	0.36696073	0.038833037	0.0443867286
SAG	0.36693567	0.03884738	0.0443867286
VPS36	0.366538	0.039075512	0.0446198474
MAK16	0.3664886	0.039103914	0.0446247502
TMEM9	0.36639544	0.039157543	0.0446402137
GUCY1B3	0.3663813	0.039165698	0.0446402137
GREB1	0.3662904	0.03921809	0.0446467613
TOX	0.36625534	0.039238308	0.0446467613
MYH10	0.36624217	0.039245907	0.0446467613
CDKL3	0.36620402	0.039267924	0.0446467613
LRDD	0.36602977	0.039368622	0.0447337749
GALNTL2	0.36588565	0.03945207	0.0448010930
ZMYM2	0.3657353	0.03953927	0.0448549609
C20orf85	0.3657203	0.039547972	0.0448549609
ACCS	0.3655969	0.039619677	0.0448941279
SCARB2	0.3655774	0.039631013	0.0448941279
MAP3K9	0.36550796	0.039671414	0.0449124081
LFNG	0.36525914	0.039816454	0.0450215756

ART3	0.36525908	0.039816488	0.0450215756
GOLGA3	0.365156	0.039876703	0.0450489468
IPW	0.36513433	0.03988937	0.0450489468
IKZF2	0.36493358	0.040006872	0.0451540976
CENPJ	0.36487842	0.04003921	0.0451630577
C11orf17	0.36482352	0.040071413	0.0451718547
NAP1L5	0.36477724	0.040098578	0.0451749652
LMF1	0.36473173	0.040125303	0.0451775766
ARMC10	0.36462244	0.04018954	0.0452061746
DBI	0.36460543	0.040199548	0.0452061746
KLHDC5	0.3644843	0.04027085	0.0452588606
RHCG	0.3644221	0.040307507	0.0452725701
GALNT1	0.36436734	0.040339813	0.0452813789
FUBP1	0.36426508	0.04040017	0.0453216453
CHCHD8	0.36406407	0.04051904	0.0454274640
SPG11	0.36393666	0.040594526	0.0454633733
NMRAL1	0.36392337	0.04060241	0.0454633733
CNBP	0.36387244	0.04063262	0.0454633733
SLC4A7	0.363821	0.040663153	0.0454633733
PUS1	0.36377236	0.04069204	0.0454633733
CEP70	0.36372066	0.04072277	0.0454633733
EIF2B3	0.36372027	0.040723	0.0454633733
ARPP-19	0.36361587	0.040785093	0.0455052484
PSPH	0.36355424	0.040821787	0.0455187516
NCALD	0.3634456	0.04088653	0.0455516622
LRRC8A	0.36342216	0.04090052	0.0455516622
SEC22C	0.3631484	0.041064087	0.0456458195
DOC2A	0.36310336	0.041091044	0.0456458195
MACROD1	0.3630874	0.04110061	0.0456458195
PIGN	0.36308214	0.04110375	0.0456458195
PPAP2A	0.3630014	0.041152127	0.0456458195
ABHD12	0.3629778	0.04116628	0.0456458195
AGTR1	0.3629627	0.04117534	0.0456458195
DCP1A	0.3629348	0.04119207	0.0456458195
CLOCK	0.3629099	0.041207004	0.0456458195
C11orf2	0.36278868	0.04127981	0.0456707270
EPHB6	0.36275786	0.041298334	0.0456707270
TFB2M	0.36274925	0.04130351	0.0456707270
C6orf70	0.36268276	0.041343503	0.0456777308
ENAH	0.36262625	0.041377515	0.0456777308
KATNB1	0.36255184	0.04142234	0.0456777308
WDR75	0.36248305	0.041463807	0.0456777308
RBMS3	0.36244172	0.041488744	0.0456777308
WDR79	0.3624292	0.0414963	0.0456777308
TMEM147	0.36238965	0.04152017	0.0456777308

FBL	0.3623774	0.041527566	0.0456777308
BBX	0.36237016	0.04153194	0.0456777308
SLC25A22	0.36229482	0.04157746	0.0457006404
F13A1	0.36217007	0.041652918	0.0457533954
LOC285370	0.3620936	0.04169923	0.0457533954
Gcom1///GRINL1A	0.36209297	0.04169961	0.0457533954
PEX6	0.3617625	0.041900225	0.0458949185
BCKDHB	0.36175954	0.041902017	0.0458949185
AP4S1	0.36175796	0.041902978	0.0458949185
PRDM6	0.36157352	0.042015288	0.0459512422
NTRK2	0.36156946	0.042017758	0.0459512422
MRPL40	0.36154935	0.04203002	0.0459512422
SSBP1	0.36151052	0.042053703	0.0459512422
EIF2B2	0.3612349	0.042222098	0.0461080256
RPL32	0.36101678	0.04235575	0.0462267059
ALS2CR8	0.3608978	0.04242879	0.0462669910
PCSK7	0.36087525	0.042442653	0.0462669910
SMARCA4	0.36082876	0.04247123	0.0462708105
ALS2CR4	0.36076978	0.042507507	0.0462708105
PRCC	0.3607476	0.04252115	0.0462708105
KIAA1522	0.3606019	0.042610895	0.0463317908
CTNND1	0.36057538	0.04262725	0.0463317908
NRP2	0.36043105	0.04271633	0.0464013655
LRP6	0.3602242	0.04284426	0.0465130353
DNAJC6	0.36016348	0.04288186	0.0465265667
MPP5	0.36010054	0.042920876	0.0465416177
SLC25A32	0.3600312	0.042963896	0.0465609903
ZNF625	0.3599533	0.043012243	0.0465861099
CCT8	0.35971886	0.043158066	0.0466314714
CPEB1	0.3596859	0.0431786	0.0466314714
TRAPPC2	0.35962102	0.043219037	0.0466314714
ZDHHC1	0.35959724	0.043233868	0.0466314714
ZNRF1	0.3595959	0.043234706	0.0466314714
C1orf21	0.3595883	0.043239444	0.0466314714
LTK	0.35951337	0.0432862	0.0466314714
ADCY3	0.3594929	0.043298982	0.0466314714
NUP88	0.35947087	0.04331274	0.0466314714
ZNF84	0.3594538	0.043323405	0.0466314714
PRKCI	0.35944125	0.043331243	0.0466314714
BEX5	0.35922837	0.04346442	0.0467340834
WNK2	0.35919088	0.043487906	0.0467340834
TMEM126A	0.35914302	0.043517906	0.0467340834
AIFM2	0.35912758	0.043527585	0.0467340834
PRNP	0.3588847	0.043680113	0.0468605101
BTK	0.35885948	0.04369597	0.0468605101

PCGF6	0.358807	0.043728992	0.0468687691
CAPS	0.3586718	0.043814145	0.0469066484
DHX9	0.35863432	0.043837782	0.0469066484
CROP	0.35857216	0.043877006	0.0469066484
DOCK7	0.3585649	0.043881577	0.0469066484
HSPD1	0.35854992	0.04389104	0.0469066484
RRN3	0.3584436	0.043958206	0.0469513210
ZFHX3	0.35838357	0.043996155	0.0469647537
USP35	0.3583363	0.04402606	0.0469695891
KPNB1	0.3582358	0.044089694	0.0470103823
CROP	0.3581232	0.044161074	0.0470593828
MUC13	0.35807168	0.04419377	0.0470671279
PCA3	0.35787866	0.044316437	0.0471350505
MEIS1	0.3578531	0.044332687	0.0471350505
INVS	0.35785115	0.04433394	0.0471350505
MRPL12	0.3577712	0.044384845	0.0471620827
CA6	0.357627	0.044476762	0.0472326371
HOOK1	0.3575286	0.044539582	0.0472336187
IARS	0.35750815	0.044552643	0.0472336187
SPIN1	0.35750565	0.04455424	0.0472336187
ANGEL2	0.35702038	0.044865146	0.0475172044
PKD2L2	0.35691422	0.04493339	0.0475172044
MTCP1	0.35688254	0.04495377	0.0475172044
AGMAT	0.35687673	0.04495751	0.0475172044
RPL36	0.35687387	0.044959355	0.0475172044
SMYD3	0.35684067	0.044980723	0.0475172044
IDH3G	0.35680118	0.045006152	0.0475172044
YDD19	0.3567127	0.04506317	0.0475172044
IFT80	0.3566975	0.04507297	0.0475172044
IKBKE	0.356689	0.04507845	0.0475172044
LRP5L	0.3565183	0.045188647	0.0476062525
VASH1	0.35643098	0.0452451	0.0476386121
DSCR6	0.35631022	0.045323264	0.0476740554
ZNF673	0.356286	0.04533896	0.0476740554
NPM3	0.35625964	0.04535603	0.0476740554
GTF2A1L	0.35611072	0.045452625	0.0477296183
C3orf22	0.35609865	0.045460463	0.0477296183
ABCC1	0.3559327	0.045568306	0.0478122602
CCDC42	0.35589808	0.045590837	0.0478122602
B3GAT3	0.35583153	0.04563416	0.0478305947
DKFZP547J0410	0.35569993	0.045719936	0.0478735144
ABCB7	0.35568935	0.045726836	0.0478735144
ADAM17	0.35564244	0.045757446	0.0478784808
DAP3	0.3554556	0.045879513	0.0479609424
SYTL2	0.35540676	0.04591147	0.0479609424

NRIP1	0.35540292	0.045913987	0.0479609424
LOC100128844	0.35530508	0.045978054	0.0480007772
CCDC41	0.35524052	0.046020363	0.0480178647
FBXO21	0.35511106	0.046105307	0.0480405188
LOC283070	0.35509464	0.04611609	0.0480405188
ANXA2	0.35506928	0.04613275	0.0480405188
REPIN1	0.35504928	0.04614589	0.0480405188
DNAJC7	0.35492972	0.04622451	0.0480953165
LOC729085	0.35484728	0.046278775	0.0481199201
C8B	0.35481483	0.04630015	0.0481199201
C6orf10	0.35462278	0.046426818	0.0482146258
ST3GAL5	0.35459772	0.04644337	0.0482146258
SLC25A23	0.35440356	0.04657175	0.0483044706
GOLGA6B	0.3543879	0.046582107	0.0483044706
C17orf81	0.35424778	0.046674956	0.0483736526
ATP2B1	0.35408208	0.046784934	0.0484038099
SH2D3A	0.35404396	0.046810262	0.0484038099
TNRC6C	0.3540332	0.046817414	0.0484038099
SRGAP3	0.35402375	0.046823695	0.0484038099
LANCL2	0.35393888	0.046880145	0.0484038099
IDI2	0.3539362	0.04688193	0.0484038099
KIF3A	0.35390836	0.046900455	0.0484038099
DUSP26	0.35388914	0.046913255	0.0484038099
PIP4K2B	0.35382622	0.046955157	0.0484200533
LOC283112	0.35357466	0.047123004	0.0485660804
SLC35F2	0.3535155	0.047162544	0.0485797824
LOC153682	0.35341603	0.047229096	0.0486212774
MORF4L2	0.35329822	0.04730801	0.0486661321
NAE1	0.3532725	0.04732525	0.0486661321
RPS25	0.3531688	0.047394823	0.0487045495
INHBC	0.3531384	0.047415234	0.0487045495
RNF115	0.35296085	0.047534563	0.0487682151
ETAA1	0.35290793	0.047570184	0.0487682151
SGCD	0.35285753	0.04760412	0.0487682151
EFEMP2	0.35284823	0.047610387	0.0487682151
C15orf44	0.35280788	0.047637578	0.0487682151
ODF2L	0.35277858	0.047657326	0.0487682151
SNRPC	0.35277218	0.047661643	0.0487682151
ZCWPW1	0.35267344	0.047728255	0.0487709301
GIPC1	0.35262278	0.047762465	0.0487709301
CDKL2	0.35261863	0.047765262	0.0487709301
SAMD4B	0.35261208	0.04776969	0.0487709301
TAP2	0.35253358	0.04782274	0.0487981763
CAMK4	0.35230935	0.04797453	0.0489260909
GSTA3	0.35223597	0.04802428	0.0489498581

DUSP5	0.35217947	0.048062623	0.0489619786
LOC388279	0.35210633	0.048112288	0.0489856134
UBE2D2	0.35203993	0.048157413	0.0489937478
DUSP9	0.3520167	0.048173215	0.0489937478
NOL1	0.3519155	0.04824206	0.0490317404
WDSOF1	0.35188395	0.04826355	0.0490317404
LOC100128822	0.35178465	0.048331216	0.0490735495
CLIP3	0.35171458	0.048379004	0.0490914268
HNRNPC	0.35168108	0.048401866	0.0490914268
PRDX4	0.35158876	0.04846492	0.0491218319
KRT76	0.3515595	0.04848492	0.0491218319
CDC25A	0.35137194	0.04861325	0.0491995638
LOC149837	0.35136968	0.048614804	0.0491995638
RING1	0.3511995	0.04873148	0.0492802517
TOM1L1	0.35117576	0.04874778	0.0492802517
PRKRIR	0.35098165	0.048881173	0.0493595467
EBF4	0.35097	0.04888919	0.0493595467
PIP5K1A	0.3509204	0.048923325	0.0493595467
CAMLG	0.35090652	0.048932884	0.0493595467
PABPC1	0.35084805	0.048973158	0.0493732655
SLC31A1	0.35072717	0.0490565	0.0493920709
ABCG8	0.35071367	0.049065817	0.0493920709
TMED6	0.3507049	0.049071863	0.0493920709
PRSS3	0.35052055	0.04919922	0.0494933458
XTP3TPA	0.35034198	0.049322836	0.0495907493
FOSL1	0.35027957	0.049366094	0.0496072964
BTN3A3	0.3501343	0.049466897	0.0496816204
TMEM66	0.3500639	0.049515825	0.0497037918
SRY	0.34999028	0.049567014	0.0497282075
NCR1	0.34983832	0.049672816	0.0498073578
LRRC8C	0.3497088	0.049763136	0.0498616754
IFRD2	0.3496651	0.04979363	0.0498616754
TCTN1	0.3496448	0.0498078	0.0498616754
LARS	0.3495425	0.04987929	0.0499062518
PDE4DIP	0.34937584	0.04999591	0.0499959100

Supplemental Table S2: table of TCF7L2 distal and proximal best epigenetic events for genes candidates which were found positively correlated with the number of blasts in CD34+ cells from CML patients during blast crisis phase: table of best TCF7L2 genomic intervals (signal CHIP-seq > 200) found in distal and proximal regions (distances from Transcription Starting Sites) corresponding to genes candidates correlated to blast number during CML blast crisis (R Pearson values and p-values). Transcription Factor genes candidates are notified in the table.

chr	TCF4_start	TCF4_end	signal	Gene Symbol	distance	Transcription Factors	Blast count R values	Blast count p Values
chr19	10305569	10305907	450.32554	EIF3G	75139		0.59027606	0.000376
chr19	49141450	49141852	375.35759	CA11	-7800		0.41806045	0.017264744
chr19	49141450	49141852	375.35759	RPL18	18976		0.51381284	0.002630048
chr22	19466616	19467056	374.50703	MRPL40	46801		0.36154935	0.04203002
chr3	169939803	169940274	373.91226	PRKCI	-181		0.35944125	0.043331243
chr15	67546924	67547215	364.34237	SMAD3	88577	YES	0.44648388	0.010419707
chr19	6737200	6737684	339.82102	SH2D3A	-30081		0.35404396	0.046810262
chr19	50380707	50381013	334.90063	ATF5	-52600	YES	0.479969	0.005435862
chr17	34135974	34136524	326.43591	TAF15	-209		0.52275586	0.002143804
chr19	12912570	12912925	325.62229	ASNA1	64442		0.37640297	0.03372581
chr19	12912570	12912925	325.62229	DNASE2	-79588		0.4660892	0.007173875
chr19	12912570	12912925	325.62229	HOOK2	26313		0.43103805	0.013780039
chr4	109541495	109541747	314.80346	RPL34	-127		0.5116218	0.002762821
chr14	39644153	39644448	314.74798	CTAGE5	-91201		0.37483573	0.034533586
chr21	44394082	44394399	309.21457	WDR4	94541		0.4080554	0.02042659
chr19	50268665	50269061	299.0367	PRMT1	88455		0.6539978	0.0000492
chr11	576120	576466	298.74362	HRAS	40743		0.48555344	0.004846099
chr10	103578177	103578519	296.24212	NPM3	35178		0.35625964	0.04535603
chr6	33290687	33291090	295.60376	PFDN6	33477		0.3822687	0.030837486
chr6	33290687	33291090	295.60376	RPS18	51037		0.58515626	0.000435
chr9	130538672	130538928	293.47431	CDK9	-9504		0.41666475	0.01767969
chr1	11072393	11072752	291.35245	EXOSC10	-87395		0.5415882	0.00136824
chr1	11072393	11072752	291.35245	SRM	-47519		0.64160264	0.0000757
chr16	21964309	21964639	278.7952	UQCRC2	-134		0.39231512	0.02636025
chr7	150076138	150076402	276.87173	REPIN1	8013	YES	0.35504928	0.04614589
chr17	61926266	61926642	268.96492	CD79B	-83250		0.4330443	0.013297985
chr17	36956136	36956423	266.17314	PIP4K2B	121		0.35382622	0.046955157
chr17	36956136	36956423	266.17314	RPL23	-53774		0.37958407	0.03213335
chr19	50354200	50354502	263.29853	ATF5	-78048	YES	0.479969	0.005435862
chr16	29874543	29874908	259.4781	PRRT2	51317		0.45568848	0.00876792
chr19	50179642	50180050	256.61887	PRMT1	-562		0.6539978	0.0000492
chr2	86332976	86333346	254.07872	LOC90784	82170		0.45522225	0.008845891

chr9	127533598	127533946	251.25046	ARPC5L	-97711		0.44184017	0.011348072
chr9	127533598	127533946	251.25046	OLFML2A	-29380		0.36930948	0.037507858
chr9	127533598	127533946	251.25046	RPL35	-90468		0.4116153	0.01925078
chr19	36134376	36134689	248.57521	TMEM147	98031		0.36238965	0.04152017
chr19	12900780	12901048	248.3907	ASNA1	52609		0.37640297	0.03372581
chr19	12900780	12901048	248.3907	DNASE2	-91421		0.4660892	0.007173875
chr19	12900780	12901048	248.3907	HOOK2	14480		0.43103805	0.013780039
chr19	14606997	14607277	248.23904	GIPC1	176		0.35262278	0.047762465
chr19	45909600	45909953	247.8102	ERCC1	-17044		0.49321467	0.004126855
chr12	54019874	54020253	243.46428	ATP5G2	-50046		0.4202213	0.016638322
chr1	32757355	32757617	243.32755	EIF3I	69516		0.45764795	0.008446557
chr1	32757355	32757617	243.32755	FAM167B	44669		0.51600873	0.002502568
chr19	19774483	19774733	242.64564	PBX4	44883	YES	0.46164408	0.007822037
chr6	31632647	31632899	231.79271	DDAH2	-65269		0.65564245	0.0000464
chr14	75348433	75348674	230.87851	DLST	-40		0.3722392	0.035906322
chr6	30524210	30524469	230.57903	GNL1	-1032		0.54002655	0.00142156
chr6	30524210	30524469	230.57903	PRR3	-146	YES	0.4694953	0.006708687
chr9	127624189	127624432	228.60298	ARPC5L	-7173		0.44184017	0.011348072
chr9	127624189	127624432	228.60298	OLFML2A	61158		0.36930948	0.037507858
chr9	127624189	127624432	228.60298	RPL35	70		0.4116153	0.01925078
chr4	104119518	104119807	226.00318	BDH2	98638		0.54186225	0.001359066
chr6	30034819	30035070	224.37132	TRIM31	-45923		0.37166384	0.0362164
chr6	31670636	31671064	224.3037	DDAH2	-27192		0.65564245	0.0000464
chr6	31670636	31671064	224.3037	VARS	-92862		0.43749833	0.012278148
chr1	46768947	46769368	223.41927	LRRC41	119		0.4716663	0.006425805
chr7	16685461	16685795	222.56786	BZW2	-130		0.41709432	0.017551104
chr12	52463501	52463817	222.13083	GRASP	62931		0.44398072	0.010911832
chr5	71615965	71616265	221.59779	MRPS27	31		0.51331604	0.002659658
chr5	133706838	133707158	221.37625	CDKL3	4233		0.36620402	0.039267924
chr2	44223086	44223457	219.35042	LRPPRC	127		0.58917826	0.000388
chrX	48755313	48755590	218.34625	PQBP1	-323		0.5977373	0.000303
chr17	49337462	49337835	216.80882	NME2	94853		0.52990484	0.001813295
chr14	21571936	21572244	208.32852	NDRG2	78905		0.41468453	0.01828261
chrX	118708327	118708566	207.74548	NKRF	-18667	YES	0.5068905	0.003069466
chr19	12848013	12848374	207.10944	ASNA1	-112		0.37640297	0.03372581
chr19	12848013	12848374	207.10944	HOOK2	-38241		0.43103805	0.013780039
chr11	57434893	57435229	205.83573	CTNND1	-94172		0.4238849	0.01561954
chr11	57434893	57435229	205.83573	SERPING1	69357		0.43178785	0.013598197
chr11	57434893	57435229	205.83573	UBE2L6	99881		0.606564	0.000233
chrX	118699365	118699592	202.53175	NKRF	-27635	YES	0.5068905	0.003069466

Supplemental Table S3: table of proximal genomic intervals with promoter prediction for TCF7L2 program active during CML blast crisis: HG19 genomic coordinates for TCF7L2 CHIP-sequencing binding intervals in proximal promoter regions are described in 3 first columns (chromosome, start and end), gene symbols of predict nearest genes from the intervals by BETA algorithm pipeline, distances in pair of bases (pb) from the Transcription Starting Sites (TSS).

chromosome	start (pb_HG19)	end (pb_HG19)	Gene_Symbol	distance from TSS (pb)
chr3	52009004	52009288	ABHD14B	500
chr6	160183372	160183656	ACAT2	4
chr6	143771860	143772144	ADAT2	161
chr6	161694294	161694578	AGPAT4	-671
chr1	46016501	46016785	AKR1A1	189
chr10	73975322	73975606	ASCC1	-735
chr10	73975322	73975606	ASCC1	-403
chr10	73975322	73975606	ASCC1	-1428
chr19	12848013	12848374	ASNA1	-112
chr2	176032772	176033056	ATF2	-20
chr21	27107196	27107480	ATP5J	-627
chr16	28833855	28834139	ATXN2L	-371
chr16	87984429	87984713	BANP	-466
chr7	16685461	16685795	BZW2	-130
chr21	30445914	30446198	CCT8	-62
chr7	92464759	92465043	CDK6	-1040
chr5	133706838	133707158	CDKL3	260
chr1	11865887	11866171	CLCN6	-123
chr1	47797691	47797975	CMPK1	-1635
chr3	149470273	149470557	COMMD2	129
chr9	131085135	131085419	COQ4	487
chr9	131084729	131084830	COQ4	-11
chr8	67974341	67974563	CSPP1	-2150
chr14	39736367	39736518	CTAGE5	115
chr15	44719325	44719609	CTDSPL2	-111
chr1	155658537	155658905	DAP3	-160
chr1	155658537	155658905	DAP3	-163
chr3	53381508	53381792	DCP1A	-4
chr6	30851502	30851786	DDR1	-670
chr6	30851502	30851786	DDR1	-682
chr6	30851502	30851786	DDR1	-1112
chr6	30851502	30851786	DDR1	-216
chr12	113623303	113623551	DDX54	143
chr12	123237222	123237506	DENR	-3
chr14	75348433	75348674	DLST	-40
chr1	44678970	44679254	DMAP1	-12
chr1	221915248	221915532	DUSP10	-128

chr7	2393667	2394544	EIF3B	-368
chr11	8008581	8008865	EIF3F	-143
chr22	29663947	29664313	EWSR1	133
chr7	121036303	121036587	FAM3C	23
chr14	45603448	45603675	FKBP3	-171
chr11	65667843	65668054	FOSL1	-49
chr1	78445137	78445421	FUBP1	390
chr19	14606997	14607277	GIPC1	176
chr19	48248577	48248861	GLTSCR2	-73
chr20	57464037	57464321	GNAS	-2246
chr20	57463832	57464116	GNAS	-205
chr20	57464037	57464321	GNAS	0
chr20	57463832	57464116	GNAS	-2451
chr6	30524210	30524469	GNL1	-1032
chr5	89854329	89854552	GPR98	-176
chr7	73868027	73868311	GTF2IRD1	50
chrX	153237066	153237350	HCFC1	389
chrX	153236438	153236620	HCFC1	-290
chr6	114292305	114292646	HDAC2	116
chr5	137911477	137911761	HSPA9	301
chr19	55791318	55791602	HSPBP1	-291
chr19	55791318	55791602	HSPBP1	235
chr9	95055847	95056131	IARS	-49
chr5	145562087	145562371	LARS	-65
chr3	45429941	45430225	LARS2	9
chr16	1020809	1021093	LMF1	-33
chr5	43065002	43065286	LOC648987	-2397
chr11	58345450	58345734	LPXN	-47
chr2	44223086	44223457	LRPPRC	127
chr1	46768947	46769368	LRRC41	119
chr9	131644229	131644513	LRRC8A	-19
chr9	131644229	131644513	LRRC8A	-409
chr6	5260586	5260870	LYRM4	-455
chrX	54835341	54835459	MAGED2	-92
chrX	43513907	43514172	MAOA	-115
chr12	112279647	112279931	MAPKAPK5	-242
chr15	42066520	42066804	MAPKBP1	31
chr15	42066297	42066581	MAPKBP1	-192
chr5	66300368	66300480	MAST4	-23
chr5	66299891	66300071	MAST4	-466
chrX	119737657	119737941	MCTS1	56
chrX	119737657	119737941	MCTS1	-752
chr5	88179334	88179618	MEF2C	193
chr2	66660666	66660797	MEIS1	-1800
chr2	66662093	66662267	MEIS1	-351

chr1	54665745	54666029	MRPL37	48
chr22	19419895	19420179	MRPL40	2
chr22	19418471	19418755	MRPL40	-1422
chr22	19419179	19419463	MRPL40	-714
chr5	71615965	71616265	MRPS27	31
chr11	47599037	47599321	NDUFS3	-1382
chr17	49230763	49231047	NME1	-14
chr17	49230963	49231247	NME1	186
chr3	23987316	23987600	NR1D2	-153
chr8	110346322	110346606	NUDCD1	114
chr4	57301880	57302164	PAICS	108
chr4	57301880	57302164	PAICS	-246
chr10	70091201	70091485	PBLD	-1341
chr11	117103075	117103359	PCSK7	-24
chr7	92158004	92158288	PEX1	301
chr5	139682764	139683048	PFDN1	217
chr17	36955667	36955823	PIP4K2B	-413
chr17	36956136	36956423	PIP4K2B	121
chr1	154909139	154909826	PMVK	-2
chr14	74181118	74181402	PNMA1	132
chr16	22308442	22308726	POLR3E	-111
chr4	57301880	57302164	PPAT	220
chrX	48754449	48754756	PQBP1	-1172
chrX	48755313	48755590	PQBP1	-323
chrX	48755313	48755590	PQBP1	257
chrX	48754449	48754756	PQBP1	-592
chrX	48755313	48755590	PQBP1	239
chrX	48754449	48754756	PQBP1	-610
chrX	48755313	48755590	PQBP1	-87
chrX	48754449	48754756	PQBP1	-936
chr1	146641945	146642229	PRKAB2	-2081
chr7	151329382	151329587	PRKAG2	140
chr7	151329382	151329587	PRKAG2	-220
chr3	169939803	169940274	PRKCI	-181
chr19	50179642	50180050	PRMT1	-562
chr6	30524210	30524469	PRR3	-146
chr11	448245	448529	PTDSS2	-1892
chr2	120517182	120517466	PTPN4	118
chr6	107077310	107077594	QRSL1	12
chr20	32581408	32581689	RALY	91
chr16	24550537	24550792	RBBP6	-243
chr5	145582701	145582985	RBM27	-319
chr1	163291470	163291754	RGS5	31
chrX	53449168	53449330	RIBC1	-555
chrX	53449312	53449596	RIBC1	-350

chr6	35435960	35436244	RPL10A	-75
chr4	109541495	109541747	RPL34	-127
chr4	109541495	109541747	RPL34	-100
chr9	127624189	127624432	RPL35	70
chr5	40835239	40835523	RPL37	-6
chr19	49999313	49999597	RPS11	-166
chr2	3622645	3622929	RPS7	-65
chr3	127842689	127842973	RUVBL1	160
chrX	134975657	134975941	SAGE1	15
chr11	65728946	65729230	SART1	-71
chr3	42641791	42642075	SEC22C	-639
chrX	118827132	118827278	SEPTIN6	-128
chrX	118827346	118827447	SEPTIN6	63
chr4	77870755	77870892	SEPTIN11	-71
chr19	48281642	48281962	SEPW1	-39
chr11	47429879	47430163	SLC39A13	-24
chr6	34725024	34725308	SNRPC	296
chr7	141437850	141438134	SSBP1	-128
chr7	141437850	141438134	SSBP1	-414
chr11	57103064	57103173	SSRP1	-233
chr1	44173094	44173378	ST3GAL3	33
chr20	2082184	2082468	STK35	-201
chr20	2082712	2083300	STK35	479
chr17	34135974	34136524	TAF15	-209
chr5	145826483	145826767	TCERG1	-247
chr15	57511384	57511668	TCF12	-128
chr6	160210555	160210839	TCP1	-38
chr2	242576860	242577137	THAP4	273
chr7	108210041	108210325	THAP5	-29
chr7	108210041	108210325	THAP5	286
chr14	58894367	58894651	TIMM9	177
chr11	85358910	85359194	TMEM126A	90
chr12	72080029	72080313	TMEM19	294
chr1	211517574	211517858	TRAF5	-1989
chr12	120884049	120884333	TRIAP1	-24
chr9	131084729	131084830	TRUB2	82
chr20	62580553	62580837	UCKL1	-1832
chr17	73781176	73781460	UNK	399
chr13	115046698	115046982	UPF3A	-218
chr16	21964309	21964639	UQCRC2	-134
chr2	85829691	85829975	USP39	-131
chr6	31763560	31763844	VARS	-10
chr3	51533251	51533535	VPRBP	-625
chr15	62352616	62352900	VPS13C	94
chr6	110500899	110500996	WASF1	-260

chr6	110501036	110501320	WASF1	-29
chr19	984246	984486	WDR18	39
chr1	111991848	111992132	WDR77	160
chr22	20116780	20117064	ZDHHC8	-2441
chr22	20118209	20118493	ZDHHC8	-1012
chr10	80828525	80828809	ZMIZ1	-124
chr11	3400207	3400491	ZNF195	-103
chr19	19976549	19976833	ZNF253	-22
chr19	54024059	54024343	ZNF331	25
chr3	179040616	179040755	ZNF639	-93
chr3	179040616	179040755	ZNF639	-865

Supplemental Table S4: stem cell signature of the CML blast crisis promoted by TCF7L2/MYC chromatin proximal binding: table of CML blast crisis genes enriched in stem cell functionalities and regulated by TCF7L2/MYC cooperation; table presents for each gene candidates their respective gene symbol, gene number ID, Pearson coefficient of correlation to the blast number (R), raw p-values and False Discovery Rate (FDR) corrected q-values

Gene symbols	gene_ID	R values	p values	FDR q values
BZW2	28969	0.41709432	0.017551104	0.0304471354301781
EIF3B	8662	0.42320132	0.015805574	0.0291235695373134
HDAC2	3066	0.36762974	0.0384518	0.0441527802729529
IARS	3376	0.35750815	0.044552643	0.0472336186941581
LRPPRC	10128	0.58917826	0.000388	0.00603519327731092
MAPKAPK5	8550	0.46502367	0.007324921	0.0209235011898148
NME1	4830	0.642063	7.46e-05	0.00264378679245283
POLR3E	55718	0.40725124	0.02070026	0.0327335471451751
PPAT	5471	0.39904958	0.023668619	0.0353311401362903
PRMT1	3276	0.6539978	4.92e-05	0.0024294375
RPL10A	4736	0.48262808	0.005147799	0.017555269198895
RUVBL1	8607	0.45813596	0.008368092	0.0216578729874652
SSRP1	6749	0.47750413	0.0057151	0.0186022474639719
TCERG1	10915	0.4506434	0.009643376	0.023147838746114
TIMM9	26520	0.48752746	0.004651199	0.0168151745097656
VARS	7407	0.43749833	0.012278148	0.0256260521245791
WDR77	79084	0.490223	0.004395956	0.0163568818614458
HSPA9	3313	0.49030593	0.004388297	0.0163568818614458
MRPL37	51253	0.41314816	0.018762069	0.0313264305323741
NME2	4831	0.52990484	0.001813295	0.0117534228461538
EWSR1	2130	0.5367986	0.001537559	0.0111172723007812
SNRPC	6631	0.35277218	0.047661643	0.0487682151426202

Supplemental Table S5: Table of the QRT-PCR primers used in functional test to validate the regulation of TCF7L2-MYC targets: this table described the primer selection used in QRT-PCR (FWD: forward primer, REV: reverse primer), length of the primer in pair of bases (pb), temperature melting temperature of the primer in Celsius degrees (°C), GC % percentage of guanine cytosine in the nucleotide sequence.

interest	name	length pb	temperature melting (°C)	GC %	sequence
Housekeeping	GAPDH_FWD	21	60.32	52.38	CAg CAA gAg CAC AAg Agg AAg
	GAPDH_REV	22	60.31	50.00	TCT ACA Tgg CAA CTg TgA ggA g
tested genes	RUVBL1_FWD	21	61.72	47.62	gCC AgC TAA TgA AgC CAA AgA
	RUVBL1_REV	21	61.09	47.62	ggA CCT CAT CAA CAA ACA gCA
	WDR77_FWD	20	60.59	55.00	CTT gTg TTg CTg CCT CTC CT
	WDR77_REV	20	60.41	50.00	CAA TCT gTg ATg CTg gCT Tg
	PRMT1_FWD	20	61.98	55.00	Agg Cgg AAA gCA gTg AgA Ag
	PRMT1_REV	21	61.43	52.38	ATg gAg TTg Cgg TAA gTg Agg
